# Synthesis and structure of a complex of copper(I) with l-cysteine and chloride ions containing Cu_12_S_6_ nanoclusters

**DOI:** 10.1107/S2056989021002012

**Published:** 2021-03-02

**Authors:** Amir Gizatullin, Jonathan Becker, Daut Islamov, Nikita Serov, Siegfried Schindler, Alexander Klimovitskii, Valery Shtyrlin

**Affiliations:** a A. M. Butlerov Chemistry Institute, Kazan Federal University, Kremlevskaya St., 18, Kazan, 420008, Russian Federation; b Institute of Inorganic and Analytical Chemistry, Justus-Liebig University of Giessen, Heinrich-Buff Ring 17, D-35392 Giessen, Germany; c A. E. Arbuzov Institute of Organic and Physical Chemistry, FRC Kazan Scientific Center of RAS, Arbuzov St. 8, 420088 Kazan, Russian Federation

**Keywords:** crystal structure, cysteine, copper(I), cage structure, SQUEEZE procedure, metal–organic framework

## Abstract

The title hydrated copper(I)–l-cysteine–chloride complex has a polymeric structure of composition {[Cu_16_(CysH_2_)_6_Cl_16_]·*x*H_2_O}_*n*_ [CysH_2_ = HO_2_CCH(NH_3_^+^)CH_2_S^−^ or C_3_H_7_NO_2_S], namely, poly[[tetra-μ_3_-chlorido-deca-μ_2_-chlorido-di­chlorido­hexa­kis­(μ_4_-l-cysteinato)hexa­deca­copper] polyhydrate]. The copper atoms are linked by thiol­ate groups to form Cu_12_S_6_ nanoclusters that take the form of a tetra­kis cubocta­hedron, made up of a Cu_12_ cubo-octa­hedral subunit that is augmented by six sulfur atoms that are located symmetrically atop of each of the Cu_4_ square units of the Cu_12_ cubo-octa­hedron. The six S atoms thus form an octa­hedral subunit themselves. The exterior of the Cu_12_S_6_ sphere is decorated by chloride ions and trichlorocuprate units. Three chloride ions are coordinated in an irregular fashion to trigonal Cu_3_ subunits of the nanocluster, and four trigonal CuCl_3_ units are bonded *via* each of their chloride ions to a copper ion on the Cu_12_S_6_ sphere. The trigonal CuCl_3_ units are linked *via* Cu_2_Cl_2_ bridges covalently connected to equivalent units in neighboring nanoclusters. Four such connections are arranged in a tetra­hedral fashion, thus creating an infinite diamond-like net of Cu_12_S_6_Cl_4_(CuCl_3_)_4_ nanoclusters. The network thus formed results in large channels occupied by solvent mol­ecules that are mostly too ill-defined to model. The content of the voids, believed to be water mol­ecules, was accounted for *via* reverse Fourier-transform methods using the SQUEEZE algorithm [Spek (2015 ▸). *Acta Cryst.* C**71**, 9–18]. The protonated amino groups of the cysteine ligands are directed away from the sphere, forming N—H⋯Cl hydrogen bonds with chloride-ion acceptors of their cluster. The protonated carb­oxy groups point outwards and presumably form O—H⋯O hydrogen bonds with the unresolved water mol­ecules of the solvent channels. Disorder is observed in one of the two crystallographically unique [Cu_16_(CysH_2_)_6_Cl_16_] segments for three of the six cysteine anions.

## Abstract 2

A cluster containing copper(I), l-cysteine and chloride ions was synthesized and characterized by X-ray diffraction and FTIR spectroscopy.

## Chemical context   

l-cysteine is an important proteinogenic amino acid widely distributed in living organisms (Lennarz & Lane, 2013 ▸). Copper–cysteine clusters are of inter­est as possible models of active sites of some copper-containing proteins (Kretsinger *et al.*, 2013 ▸). It is inter­esting to observe that there are no structures of copper complexes with both chloride ions and cysteine and even cystine determined by single crystal X-ray diffraction. As part of our studies in this area, we now describe the synthesis and structure of the title cluster compound.
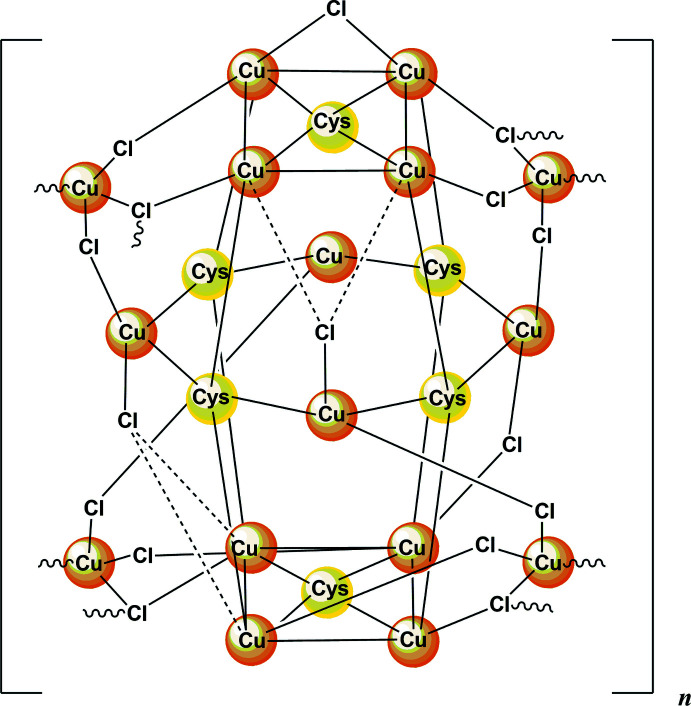


## Structural commentary   

The crystallographic analysis of the title compound revealed a complex polymeric structure of composition {[Cu_16_(CysH_2_)_6_Cl_16_]·*x*H_2_O}_*n*_, [CysH_2_ = HO_2_CCH(NH_3_^+^)CH_2_S^−^]. The copper atoms are linked by thiol­ate groups to form Cu_12_S_6_ copper thiol­ate nanoclusters (‘atlas spheres’), which have the form of a tetra­kis cubocta­hedron, made up of a Cu_12_ cubo-octa­hedral subunit that is augmented by six sulfur atoms that are located symmetrically atop of each of the Cu_4_ square units of the Cu_12_ cubo-octa­hedron. The six S atoms form an octa­hedral subunit themselves. The exterior of the Cu_12_S_6_ sphere is decorated by chloride ions and trichloro­cuprate units. Three chloride ions are irregularly coordinated to trigonal Cu_3_ subunits of the nanocluster, and four trigonal CuCl_3_-units are linked through each of their chloride ions to each one copper ion on the ‘atlas spheres’. The trigonal CuCl_3_ units are covalently connected through Cu_2_Cl_2_ bridges to equivalent units in neighboring nanoclusters. Four such connections are arranged in a tetra­hedral fashion, forming a diamond like network of Cu_12_S_6_Cl_4_(CuCl_3_)_4_ nanoclusters. The rigid diamond-like network results in large channels occupied by solvate mol­ecules, which in most cases were too poorly defined for modeling. The content of the voids, believed to be water mol­ecules, was accounted using reverse Fourier-transform methods using the SQUEEZE algorithm (Spek, 2015 ▸). The protonated amino groups of the cysteine ligands are directed away from the sphere, forming N—H⋯Cl hydrogen bonds with chloride ions of their cluster. The protonated –CO_2_H carb­oxy groups point outwards into the void and presumably form O—H⋯O hydrogen bonds with the unresolved water mol­ecules in the solvate channels (the carboxyl­ate protons are omitted in the structure).

Conclusion about the state of the carb­oxy groups is based on the following facts: (i) the FTIR spectrum confirms the presence of –CO_2_H groups and the absence of H_3_O^+^ ions in the crystal (see below); (ii) the coordination geometries observed are strongly favored by Cu^I^; (iii) the crystals of the complex are colorless, which excludes the presence of copper(II).

**Figure 1:**
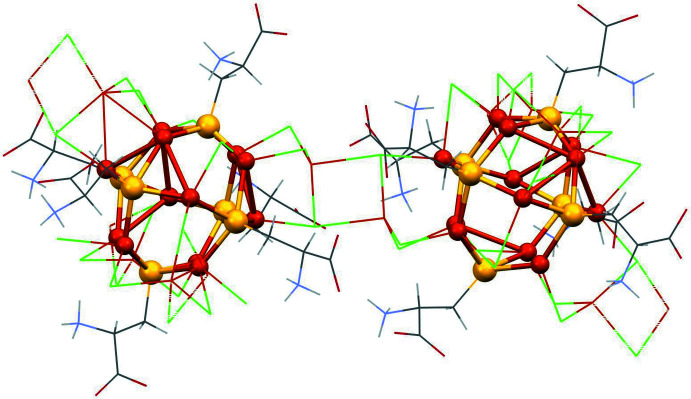
Asymmetric unit of the title compound. Orange: copper, yellow: sulfur, green: chlorine, red: oxygen, blue: nitro­gen.

Disorder is observed in one of the two crystallographically unique [Cu_16_(CysH_2_)_6_Cl_16_] clusters for three of the six cysteine ligands. The asymmetric unit consists of two Cu_12_ distorted cubo-octa­hedra (Figs. 1 ▸, 2 ▸). Almost all of the Cu—S bonds are similar in length (mean 2.25 ± 0.03 Å) except for the bonds formed by the disordered S1_1, S1_5 and S1_12 atoms, where the Cu—S bond lengths were determined with higher errors. The S—Cu—S angles are clustered in a narrow range (mean 130 ± 4°). Thus the Cu—S bonds and angles are typical for such Cu_12_S_6_ copper thiol­ate nanoclusters (see *Database survey*).

In the ‘atlas sphere’ there are four tetra­hedral copper atoms (atoms Cu17, Cu26, Cu28, Cu32 for the first core and Cu1, Cu9, Cu11, Cu16 for the second) surrounded by two μ_2_-chloride ions and one μ_3_-chloride ion (for example, Cu1 ion is surrounded by Cl1, Cl2 and Cl3 atoms), which are close to planar with copper and the μ_3_-Cl that is almost perpendicular to this imaginary plane wherein the length of Cu—μ_3_-Cl bond is longer than the others (mean 2.58 ± 0.04 Å). We note that the lengths of the other Cu—μ_3_-Cl bonds are about the same as the Cu—μ_2_-Cl lengths (mean 2.31 ± 0.04 Å) and the Cl—Cu—Cl angle in the [Cu_2_Cl_2_] units is 94.9 ± 2.4°. In addition, there are two non-bridging chloride ions: Cl28 and Cl26. The other chloride ions form μ_2_-bridges between the copper ions in the core except for μ_3_-Cl15.

The charge distribution per cage is as following: 16 positive charges of Cu^+^ ions are balanced by the negative charges of 16 chloride ions. The 12 amino acid residues occur as neutral CysH_2_ = HO_2_CCH(NH_3_^+^)CH_2_S^−^ zwitterions. The ‘atlas spheres’ in the asymmetric unit have differences regarding the presence of disorder, *viz.* three of the six cysteine mol­ecules are disordered in one ‘atlas sphere’ while the other is not disordered.

## Supra­molecular features   

**Figure 3:**
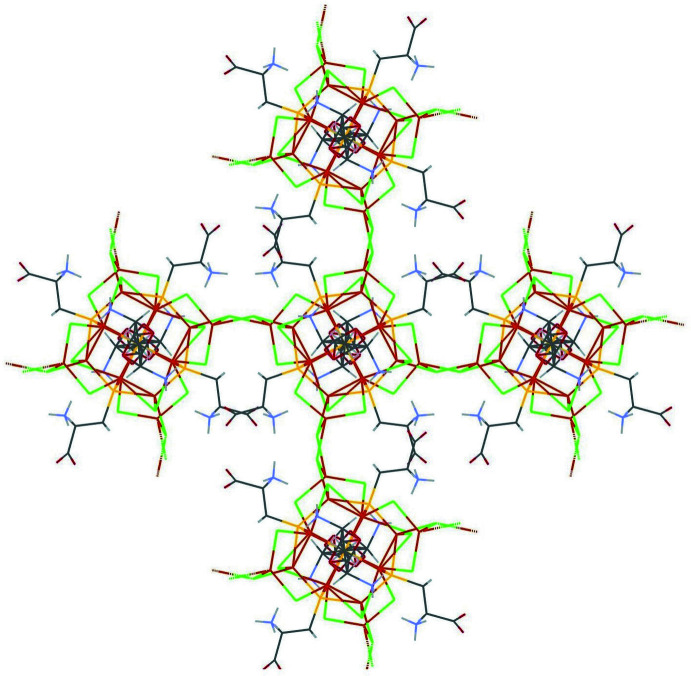
Tetra­hedral environment of ‘atlas-sphere’ of the title compound.

**Figure 4:**
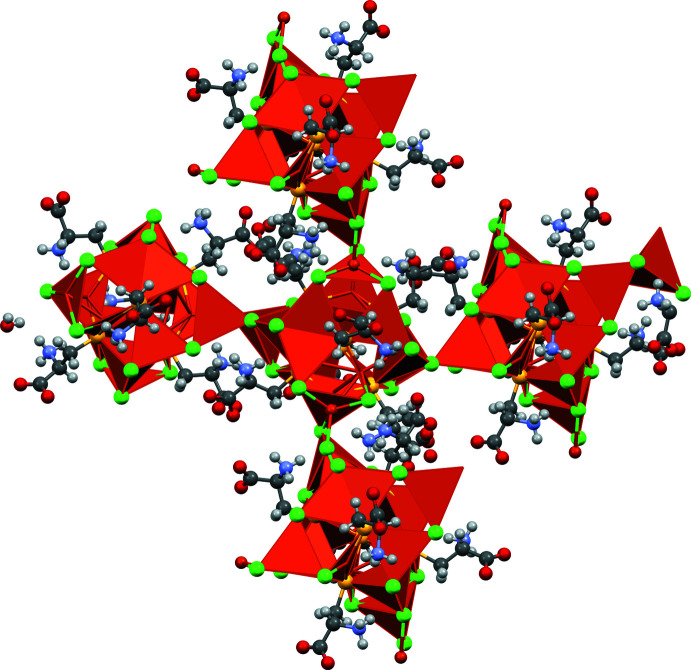
Diamond-like extended structure of the title compound.

**Figure 5:**
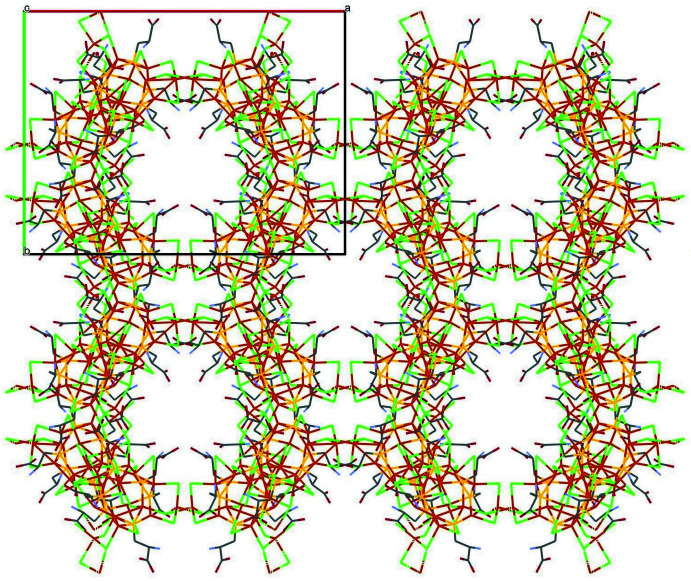
Crystal packing of the title compound viewed along *c-*axis direction.

In the structure, the ‘atlas spheres’ are linked to form a three-dimensional framework with the Cu_2_Cl_2_ linkages forming a tetra­hedral environment in each of the clusters (Fig. 3 ▸). As a result, the ‘atlas spheres’ form a distorted diamond-like structure (Fig. 4 ▸). However, it is not possible to give an exact description of the topology (O’Keeffe *et al.*, 2008 ▸). These bridges are based on the μ_3_-Cl atoms described above, with the exception of Cl15 and eight copper atoms in a distorted tetra­hedral environment (four such atoms per cage); thus, from the point of view of the coordination environment, it is more accurate to talk about Cu_2_Cl_8_ bridges. In addition, the cages are connected by a system of hydrogen bonds. Namely, two water mol­ecules (O4_6 and O3_6) act as donors for two amino groups (N1_9 and N1_6, respectively), forming N—H⋯O hydrogen bonds. In turn, the water mol­ecules are linked by hydrogen bonds. Thus, a chain of three hydrogen bonds exists between neighboring ‘atlas spheres’. The structure has voids in which there are presumably disordered water mol­ecules (Figs. 5 ▸, 6 ▸). Using *PLATON* SQUEEZE (Spek, 2015 ▸), a void was identified occupying 38.6% of the unit-cell volume for the compound. The void volume of 7685 Å^3^ contains the equivalent of 3455 electrons, corresponding to about 346 water mol­ecules. The hydrogen-bond geometry is given in Table 1 ▸.

## Database survey   

Considering copper(I) complexes with cysteine, a number of heteroligand Co^III^/Cu^I^ complexes with ethyl­enedi­amine are known where the inner sphere of Co^III^ contains two coord­in­ated ethyl­enedi­amine mol­ecules and one monoprotonated l-cysteine mol­ecule coordinated *via* nitro­gen and sulfur ([Co(en)_2_(l-CysH)]). In addition, the sulfur atom of cysteine is coordinated to the Cu^I^ atom, which is surrounded by other sulfur atoms and chloride ions ([CuCl_3_S], [CuClS_3_], [CuClS_2_]), for example, see Cambridge Structural Database (CSD; Groom *et al.*, 2016 ▸) refcodes TOHREO, XOMDEJ, XOMDIN, XOMDOT, XOMDUZ, XOMFAH (Aridomi *et al.*, 2008 ▸). A copper(II) complex with *S*-methyl-l-cysteine of composition [Cu(l-MeCys)_2_]_*n*_ (MeCysH = HO_2_CCH(NH_2_)CH_2_SCH_3_) has been characterized (Dubler *et al.*, 1986 ▸). The ligand coordin­ates to the metal ion *via* its oxygen and nitro­gen atoms and the structure is polymeric because both carb­oxy­lic groups are also coordinated to other copper(II) atoms. It should be noted that only one copper(II)–cystine complex has been synthesized, which includes 2,2′-bipyridyl as a second ligand (Seko *et al.*, 2010 ▸).

Several copper–cyste­amine (CyH = ^−^SCH_2_CH_2_NH_3_^+^) structures have been reported: {[Cu_8_Cl_6_(CyH)_6_]Cl_2_}_*n*_ (Salehi *et al.*, 1997 ▸); [Cu_13_Cl_13_(CyH)_6_·H_2_O]_*n*_ consisting of [Cu_12_S_6_Cl_12_] clusters bridged by Cl and [Cu_2_Cl_2_] units (Parish *et al.*, 1997 ▸); {[Cu_13_(CyH)_6_Br_13_]·*x*H_2_O}_*n*_ formed of [Cu_12_(CyH)_6_Br_12_] clusters, which are linked by [Cu_2_Br_2_] bridges (Prichard *et al.*, 1999 ▸), and the most recent one [Cu_3_Cl(Cy)_2_] (here cyste­amine has deprotonated amino and thio groups) where there are parallel chains [Cu_2_Cy_2_]_*n*_ connected to neighboring ones by [CuCl] links (Ma *et al.*, 2014 ▸). In addition, there are five complexes of copper(I) with cystamine (H_2_NCH_2_CH_2_S–SCH_2_CH_2_NH_2_) and bromide ligands (Louvain *et al.*, 2008 ▸). The dimethyl derivative of cyste­amine forms a complex {[Cu_17_(*R*S)_6_Cl_17_]}_*n*_ [*R*S = ^−^SCH_2_CH_2_NH(CH_3_)_2_^+^] including a [Cu_12_S_6_] cluster (Prichard *et al.*, 1999 ▸). In addition, the [Cu_6_S_12_] cluster has been found in copper–thiol systems: [Cu_12_(S*R*′)_6_Cl_12_][(Cu(*R*′SH))_6_] (*R*′ = *n*-Bu) and [H(THF)_2_]_2_[Cu_17_(S*R*′′)_6_Cl_13_(THF)_2_(*R*′′SH)_3_] (*R*′′ = CH_2_CH_2_Ph) (Cook *et al.*, 2019 ▸).

Thus [Cu_12_S_6_] clusters in copper(I) complexes with cyste­amine, a close derivative of cysteine, are stabilized with Cl^−^ or Br^−^ anions. Chloride ions also stabilize such clusters containing simple thiols. It should be noted that phosphine ligands also can stabilize a [Cu_12_S_6_] core containing just sulfur instead of thiols and forming [Cu_12_S_6_(P*R*_3_)_8_] complexes: [Cu_12_S_6_(PPh_2_Et)_8_], [Cu_12_S_6_(PEt_3_)_8_] (Dehnen *et al.*, 1994 ▸) and [Cu_12_S_6_(P_*n*_P*R*_3_)_8_] (Dehnen *et al.*, 1996 ▸). Moreover, there are some complexes containing four diphosphine ligands (Eichhöfer *et al.*, 2015 ▸, Yang *et al.*, 2014 ▸, Khadka *et al.*, 2013 ▸) with high photoluminescence quantum yields.

## Synthesis and crystallization   

Masses of 0.085 g (0.500 mmol) of CuCl_2_·2H_2_O and 0.060 g (0.50 mmol) of l-cysteine were mixed in 5 ml of water under inert conditions. A precipitate was formed, which was dissolved by adding approximately 1 ml of a 2 *M* HCl oxygen-free solution. The resulting solution was left to stand in an inert atmosphere. Colorless crystals of the title compound formed within 24 h.

**Figure 7:**
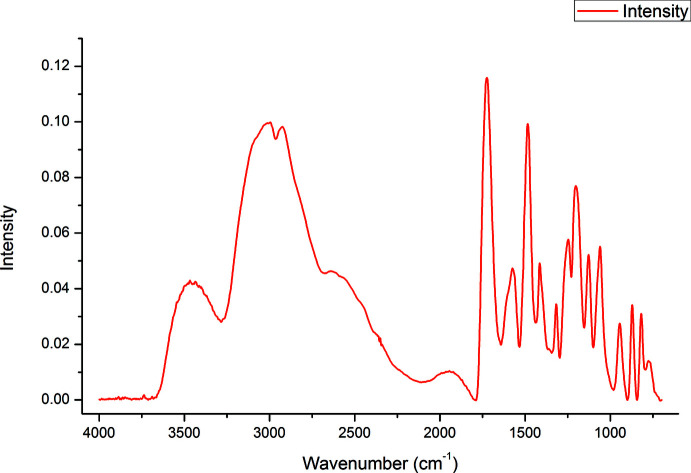
IR spectrum of the title compound.

As a result of the rapid degradation of the crystals in air, it was not possible to perform an elemental analysis. The IR spectra of the crystals were recorded using an FTIR Bruker Vertex 70 spectrometer (400–4000 cm^−1^). The IR spectrum of {[Cu_16_(CysH_2_)_6_Cl_16_]·*x*H_2_O}_*n*_ (**1**) is shown in Fig. 7 ▸, and the spectroscopic parameters are presented in Table 2 ▸ in comparison with the corresponding values for the crystal of l-cysteine hydro­chloride, l-CysH_2_·HCl (Dokken *et al.*, 2009 ▸) along with our assignment of the spectroscopic lines.

As follows from Table 2 ▸, there is a satisfactory correspondence of most bands of both crystals, **1** and l-CysH_2_·HCl. Of particular note is the almost complete coincidence of the position of the most intense line at 1201–1203 cm^−1^ for both compounds. According to Dokken *et al.* (2009 ▸), this intense band is associated with vibrations of the protonated –COOH group. In this case, the possibility of protonation of water mol­ecules instead of a carb­oxy group with the hydroxonium ion formation is practically excluded. Indeed, according to numerous experimental and calculated data for crystals and liquid phases, H_3_O^+^ ions show four broad lines in the IR spectra near 1150, 1740, 3160, and 3320 cm^−1^ (Chukanov, 2014 ▸; Yukhnevich, 1973 ▸). As follows from Fig. 7 ▸ and Table 2 ▸, no sign of these bands was detected in the spectrum of the crystal **1**. On the other hand, there is a satisfactory agreement between the vibration lines of the –NH_3_^+^ group at ∼1130, ∼1572, ∼1610, and ∼2930 cm^−1^ for compounds **1** and l-CysH_2_·HCl. Thus, according to the IR spectroscopic data, the protons in **1** are localized on the carboxyl and ammonium groups, while the thiol groups are deprotonated and bonded to copper(I).

## Refinement   

Crystal data, data collection and structure refinement details are summarized in Table 3 ▸. All non-hydrogen atoms were refined anisotropically and C—H hydrogen atoms were positioned at geometrically calculated positions (C—H = 0.99–1.00 Å, N—H = 0.91 Å) and refined using a riding model. The constraint *U*_iso_(H) = 1.2*U*_eq_(C) or 1.5*U*_eq_(N) was applied in all cases. Three of the cysteine ligands were found to be disordered over two sets of sites with refined major occupancies of 0.826 (8), 0.550 (19) and 0.657 (9). Close to one of the disordered cysteine ligands, two partially occupied water mol­ecules were found, which could not be modeled using SQUEEZE (Spek, 2015 ▸) because of their proximity to the cysteine disorder. Their occupancy was refined freely and converged to 0.55 (2) and 0.33 (2). The structure was refined with the help of similarity restraints, strong similarity restraints on anisotropic displacement parameters (Müller, 2009 ▸) and rigid bond restraints (Thorn *et al.*, 2012 ▸) on the disordered ligands. One of the partially occupied water mol­ecules was strongly restrained to have a more isotropic behavior using the ISOR instruction as implemented in *SHELXL*. The unit cell contains a significant amount of solvent, most likely a heavily disordered hydrogen-bonded network of water mol­ecules. To refine the model against the measured data, the SWAT instruction as implemented in *SHELXL* (Langridge *et al.*, 1960 ▸; Driessen *et al.*, 1989 ▸) was used. In addition, SQUEEZE (Spek, 2015 ▸) as implemented in *PLATON* (Spek, 2020 ▸) was used to model the disordered solvent in the voids of the structure. SQUEEZE identified a void centered at ∼(0 0.1 0) with a volume of 7685 Å^3^ containing the equivalent of 3455 electrons. This would correspond to about 346 water mol­ecules.

## Supplementary Material

Crystal structure: contains datablock(s) I. DOI: 10.1107/S2056989021002012/hb7953sup1.cif

Structure factors: contains datablock(s) I. DOI: 10.1107/S2056989021002012/hb7953Isup2.hkl

Supporting information file. DOI: 10.1107/S2056989021002012/hb7953Isup4.cdx

Author's response to referees comments for previous submission ZL2796. DOI: 10.1107/S2056989021002012/hb7953sup3.txt

CCDC reference: 2064015

Additional supporting information:  crystallographic information; 3D view; checkCIF report

## Figures and Tables

**Figure 2:**
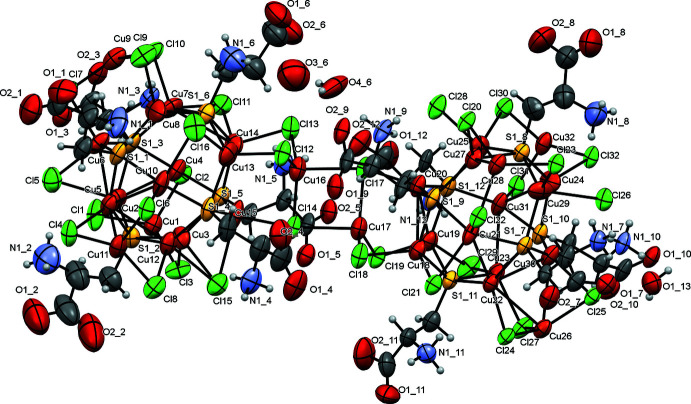
Displacement ellipsoid plot (50% probability level) of the asymmetric unit.

**Figure 6:**
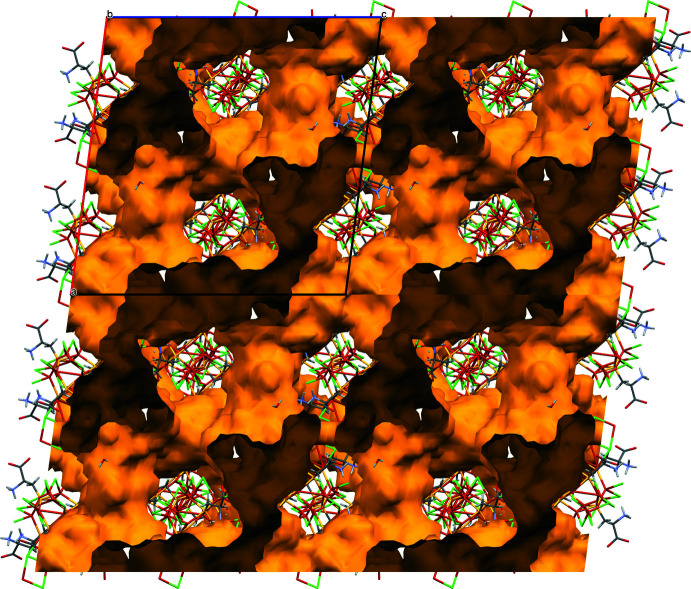
Visualization of the void space of the title compound viewed along the *b*-axis direction.

**Table 1:** Hydrogen-bond geometry (Å, °)

*D*—H⋯*A*	*D*—H	H⋯*A*	*D*⋯*A*	*D*—H⋯*A*
N1_1—H1*A*_1⋯Cl6	0.91	2.73	3.554 (19)	151
N1_1—H1*A*_1⋯Cl16	0.91	2.66	3.148 (16)	115
N1_1—H1*B*_1⋯Cl3^i^	0.91	2.81	3.42 (2)	126
N1*A*_1—H1*AA*_1⋯Cl7	0.91	2.74	3.34 (4)	125
N1*A*_1—H1*AA*_1⋯Cl9	0.91	2.54	3.28 (3)	138
N1_2—H1*A*_2⋯Cl4	0.91	2.78	3.395 (15)	126
N1_2—H1*A*_2⋯O2*A*_5^i^	0.91	2.48	3.04 (2)	120
N1_2—H1*C*_2⋯Cl5	0.91	2.19	3.101 (15)	175
N1_3—H1*A*_3⋯Cl10	0.91	2.69	3.592 (9)	171
N1_3—H1*B*_3⋯O2_3	0.91	2.13	2.605 (10)	112
N1_3—H1*C*_3⋯Cl2	0.91	2.53	3.306 (8)	143
N1_3—H1*C*_3⋯Cl11	0.91	2.68	3.228 (9)	120
N1_4—H1*B*_4⋯Cl15	0.91	2.44	3.199 (10)	141
N1_4—H1*B*_4⋯S1_4	0.91	2.82	3.300 (10)	114
N1_5—H1*B*_5⋯O2_12	0.91	2.36	3.14 (3)	144
N1_5—H1*C*_5⋯Cl11	0.91	2.76	3.46 (2)	134
N1_5—H1*C*_5⋯Cl13	0.91	2.49	3.24 (2)	139
N1*A*_5—H1*A*2_5⋯Cl2	0.91	2.74	3.46 (2)	137
N1*A*_5—H1*A*2_5⋯Cl11	0.91	2.42	2.99 (2)	121
N1*A*_5—H1*A*2_5⋯S1*A*_5	0.91	2.68	3.22 (4)	119
N1*A*_5—H1*A*3_5⋯Cl4^ii^	0.91	2.82	3.49 (3)	132
N1_6—H1*B*_6⋯O3_6	0.91	1.92	2.70 (3)	142
N1_6—H1*C*_6⋯Cl16	0.91	2.53	3.311 (16)	145
N1_6—H1*C*_6⋯S1_6	0.91	2.89	3.330 (16)	111
N1*A*_6—H1*AC*_6⋯Cl11	0.91	2.74	3.60 (7)	158
N1_7—H1*B*_7⋯Cl23	0.91	2.77	3.435 (9)	131
N1_7—H1*B*_7⋯Cl26	0.91	2.58	3.315 (9)	138
N1_7—H1*C*_7⋯O1_13	0.91	1.99	2.803 (12)	148
N1_8—H1*C*_8⋯Cl26	0.91	2.51	3.370 (12)	158
N1_8—H1*C*_8⋯S1_8	0.91	2.83	3.286 (13)	112
N1_9—H1*A*_9⋯Cl20	0.91	2.85	3.585 (9)	139
N1_9—H1*A*_9⋯O1_10^iii^	0.91	2.12	2.796 (11)	130
N1_9—H1*B*_9⋯O4_6	0.91	2.01	2.86 (3)	154
N1_9—H1*C*_9⋯Cl17	0.91	2.79	3.348 (9)	121
N1_9—H1*C*_9⋯Cl28	0.91	2.45	3.211 (9)	142
N1_10—H1*B*_10⋯Cl25	0.91	2.77	3.239 (9)	113
N1_10—H1*B*_10⋯Cl26	0.91	2.57	3.363 (8)	146
N1_10—H1*C*_10⋯O2_9^iv^	0.91	2.15	2.856 (12)	134
N1_11—H1*A*_11⋯O1_13^v^	0.91	2.08	2.971 (13)	167
N1_11—H1*C*_11⋯Cl21	0.91	2.61	3.362 (10)	141
N1_11—H1*C*_11⋯Cl24	0.91	2.61	3.235 (9)	126
N1_12—H1*A*_12⋯Cl31	0.91	2.92	3.387 (9)	113
N1_12—H1*C*_12⋯Cl29	0.91	2.44	3.316 (9)	161
O4_6—H4*B*_6⋯Cl12	0.84 (1)	2.89 (10)	3.32 (3)	114 (8)
O4_6—H4*B*_6⋯O3_6	0.84 (1)	1.76 (7)	2.51 (4)	148 (10)
O1_13—H1*A*_13⋯Cl25	0.82 (3)	2.97 (3)	3.501 (9)	125 (2)

Symmetry codes: (i) -x+{\script{1\over 2}}, y+{\script{1\over 2}}, -z+1; (ii) -x+{\script{1\over 2}}, y-{\script{1\over 2}}, -z+1; (iii) -x+{\script{1\over 2}}, y+{\script{1\over 2}}, -z; (iv) -x+{\script{1\over 2}}, y-{\script{1\over 2}}, -z; (v) -x+1, y, -z.

**Table 2:** Comparison of infrared band assignments (cm^−1^) for **1** and L-cysteine hydro­chloride, L-CysH_2_·HCl (Dokken *et al.*, 2009 ▸)

**1**	L-CysH_2_·HCl	Assignment
776 *w*	770 *w*	γ CH_2_
819 *w*	839 *w*	δ COO^−^
871 *mw*	868 *mw*	ν CC, δ COO^−^
944 *w*	929 *w*	ν CN, ν CC
1060 *mw*	1058 *mw*	ν CN, ν CC
1127 *mw*	1141 *mw*	NH_3_^+^
1203 *s*	1201 *s*	ν CO, δ OH (COOH)
1247 *mw*	1272 *w*	γ CH_2_
1317 *w*	1344 *w*	δ CH
1415 *m*	1427 *m*	δ as CH_3_, δ CH_2_
1484 *s*	1477 *sh*	–
1574 *mw*	1571 *mw*	δ as NH_3_^+^, ν as COO^−^
1601 *sh*	1619 *w*	δ as NH_3_^+^, ν as COO^−^
1724 *vs*	1743 *vs*	ν CO
1968 *w*	–	–
2641 *sh*	2645 *w*	ν CH_2_
2923 *s*	2943 *sh*	ν NH_3_^+^, ν CH_2_, ν CH_3_
3011 *s*	3051 *sh*	ν NH_3_^+^
3453 *m*	–	–

**Table 3:** Experimental details

Crystal data	
Chemical formula	[Cu_32_Cl_32_(C_3_H_6_NO_2_S)_12_]·2.68H1.97O
*M*_r_	4643.74
Crystal system, space group	Monoclinic, *C*2
Temperature (K)	100
*a*, *b*, *c* (Å)	29.4665 (14), 22.1299 (11), 28.9371 (14)
β (°)	97.3964 (14)
*V* (Å^3^)	18712.6 (16)
*Z*	4
Radiation type	Mo *K*α
μ (mm^−1^)	4.18
Crystal size (mm)	0.36 × 0.31 × 0.13

Data collection	
Diffractometer	Bruker PHOTON100
Absorption correction	Multi-scan (*SADABS*; Sheldrick, 2016 ▸)
*T*_min_, *T*_max_	0.487, 0.746
No. of measured, independent and observed [*I* > 2σ(*I*)] reflections	189540, 33050, 26149
*R*_int_	0.050
(sin θ/λ)_max_ (Å^−1^)	0.595

Refinement	
*R*[*F* ^2^ > 2σ(*F* ^2^)], *wR*(*F* ^2^), *S*	0.041, 0.114, 1.09
No. of reflections	33050
No. of parameters	1568
No. of restraints	1778
H-atom treatment	H atoms treated by a mixture of independent and constrained refinement
Δρ_max_, Δρ_min_ (e Å^−3^)	0.56, −0.74
Absolute structure	Flack *x* determined using 10959 quotients [(*I* ^+^)−(*I* ^−^)]/[(*I* ^+^)+(*I* ^−^)] (Parsons *et al.*, 2013 ▸)
Absolute structure parameter	0.030 (4)

Computer programs: *APEX3* and *SAINT* (Bruker, 2017 ▸), *SHELXT2015* (Sheldrick, 2015*a* ▸), *SHELXL2018/3* (Sheldrick, 2015*b* ▸) and *SHELXTL* (Sheldrick, 2008 ▸).

## supplementary crystallographic information

### Crystal data

**Table:**

[Cu_32_Cl_32_(C_3_H_6_NO_2_S)_12_]·2.68H1.97O	*F*(000) = 8986
*M**_r_* = 4643.74	*D*_x_ = 1.648 Mg m^−^^3^
Monoclinic, *C*2	Mo *K*α radiation, λ = 0.71073 Å
*a* = 29.4665 (14) Å	Cell parameters from 9656 reflections
*b* = 22.1299 (11) Å	θ = 2.3–27.0°
*c* = 28.9371 (14) Å	µ = 4.18 mm^−^^1^
β = 97.3964 (14)°	*T* = 100 K
*V* = 18712.6 (16) Å^3^	Block, colourless
*Z* = 4	0.36 × 0.31 × 0.13 mm

### Data collection

**Table:**

Bruker PHOTON100 diffractometer	33050 independent reflections
Radiation source: IµS micro-focus sealed tube, multilayer optics	26149 reflections with *I* > 2σ(*I*)
Detector resolution: 10.4167 pixels mm^-1^	*R*_int_ = 0.050
φ and ω scans	θ_max_ = 25.0°, θ_min_ = 1.4°
Absorption correction: multi-scan (SADABS; Sheldrick, 2016)	*h* = −35→35
*T*_min_ = 0.487, *T*_max_ = 0.746	*k* = −26→26
189540 measured reflections	*l* = −34→34

### Refinement

**Table:**

Refinement on *F*^2^	Secondary atom site location: difference Fourier map
Least-squares matrix: full	Hydrogen site location: mixed
*R*[*F*^2^ > 2σ(*F*^2^)] = 0.041	H atoms treated by a mixture of independent and constrained refinement
*wR*(*F*^2^) = 0.114	*w* = 1/[σ^2^(*F*_o_^2^) + (0.0624*P*)^2^ + 8.4715*P*] where *P* = (*F*_o_^2^ + 2*F*_c_^2^)/3
*S* = 1.09	(Δ/σ)_max_ = 0.005
33050 reflections	Δρ_max_ = 0.56 e Å^−^^3^
1568 parameters	Δρ_min_ = −0.74 e Å^−^^3^
1778 restraints	Absolute structure: Flack *x* determined using 10959 quotients [(*I*^+^)-(*I*^-^)]/[(*I*^+^)+(*I*^-^)] (Parsons *et al.*, 2013)
Primary atom site location: dual	Absolute structure parameter: 0.030 (4)

### Special details

**Table:**

Geometry. All esds (except the esd in the dihedral angle between two l.s. planes) are estimated using the full covariance matrix. The cell esds are taken into account individually in the estimation of esds in distances, angles and torsion angles; correlations between esds in cell parameters are only used when they are defined by crystal symmetry. An approximate (isotropic) treatment of cell esds is used for estimating esds involving l.s. planes.
Refinement. Bruker D8 Venture Dual IµS fixed chi instrument.

### Fractional atomic coordinates and isotropic or equivalent isotropic displacement parameters (Å^2^)

**Table:**

	*x*	*y*	*z*	*U*_iso_*/*U*_eq_	Occ. (<1)
Cu1	0.23519 (5)	0.31230 (6)	0.54032 (5)	0.0579 (4)	
Cu2	0.20172 (5)	0.44258 (8)	0.54083 (5)	0.0691 (5)	
Cu3	0.26766 (5)	0.41381 (6)	0.47398 (5)	0.0672 (5)	
Cu4	0.17391 (5)	0.36921 (6)	0.46655 (5)	0.0559 (4)	
Cu5	0.21846 (5)	0.58001 (6)	0.51074 (5)	0.0567 (4)	
Cu6	0.13091 (5)	0.53887 (6)	0.51306 (6)	0.0630 (5)	
Cu7	0.09631 (5)	0.44665 (8)	0.42588 (6)	0.0798 (5)	
Cu8	0.10651 (7)	0.57526 (9)	0.40780 (7)	0.0977 (7)	
Cu9	0.02387 (5)	0.54135 (7)	0.45601 (6)	0.0745 (5)	
Cu10	0.20700 (6)	0.61803 (6)	0.41657 (6)	0.0698 (5)	
Cu11	0.28239 (5)	0.67012 (6)	0.48669 (5)	0.0626 (5)	
Cu12	0.28192 (5)	0.54007 (6)	0.45474 (5)	0.0614 (4)	
Cu13	0.17952 (6)	0.53201 (8)	0.33686 (5)	0.0785 (5)	
Cu14	0.15793 (5)	0.40690 (7)	0.36370 (5)	0.0695 (5)	
Cu15	0.25597 (7)	0.45178 (6)	0.37674 (6)	0.0824 (6)	
Cu16	0.20923 (6)	0.43008 (6)	0.27659 (5)	0.0651 (5)	
Cl17	0.22173 (9)	0.37582 (11)	0.19939 (9)	0.0545 (7)	
Cl18	0.34231 (11)	0.45854 (12)	0.21509 (10)	0.0665 (8)	
Cl19	0.33498 (9)	0.28328 (11)	0.24565 (9)	0.0570 (7)	
Cl20	0.19600 (9)	0.48492 (10)	0.00147 (9)	0.0517 (7)	
Cl21	0.40264 (9)	0.49108 (12)	0.11204 (9)	0.0552 (7)	
Cl22	0.32955 (9)	0.54141 (10)	0.00565 (9)	0.0505 (6)	
Cl23	0.27217 (9)	0.44123 (11)	−0.09437 (9)	0.0510 (6)	
Cl24	0.48113 (8)	0.37736 (11)	0.06546 (9)	0.0475 (6)	
Cl25	0.44196 (8)	0.25954 (10)	−0.03622 (9)	0.0457 (6)	
Cl26	0.31903 (10)	0.28123 (11)	−0.09826 (9)	0.0569 (7)	
Cl27	0.46038 (9)	0.20465 (13)	0.09290 (11)	0.0633 (8)	
Cl28	0.15872 (11)	0.35724 (12)	0.07666 (11)	0.0684 (8)	
Cl29	0.36295 (11)	0.16125 (14)	0.15790 (12)	0.0724 (9)	
Cl30	0.16584 (9)	0.20464 (11)	0.03029 (10)	0.0576 (7)	
Cl31	0.26426 (9)	0.09887 (10)	0.07751 (9)	0.0544 (7)	
Cl32	0.24954 (9)	0.15419 (10)	−0.05720 (9)	0.0499 (7)	
Cu17	0.29960 (5)	0.37517 (6)	0.23092 (5)	0.0575 (4)	
Cu18	0.34600 (5)	0.40742 (6)	0.14677 (5)	0.0554 (4)	
Cu19	0.32706 (5)	0.27978 (6)	0.16614 (5)	0.0544 (4)	
Cu20	0.25792 (4)	0.35959 (5)	0.13493 (4)	0.0491 (4)	
Cu21	0.34111 (5)	0.45217 (6)	0.05047 (5)	0.0597 (5)	
Cu22	0.40555 (4)	0.36029 (5)	0.07127 (4)	0.0449 (4)	
Cu23	0.38409 (5)	0.23608 (6)	0.09839 (5)	0.0588 (4)	
Cu24	0.27759 (5)	0.23655 (6)	−0.01539 (6)	0.0726 (5)	
Cu25	0.21648 (5)	0.28572 (6)	0.05507 (5)	0.0591 (4)	
Cu26	0.47292 (4)	0.27944 (6)	0.04148 (5)	0.0530 (4)	
Cu27	0.24038 (5)	0.40504 (5)	0.03107 (5)	0.0531 (4)	
Cu28	0.26203 (5)	0.50137 (6)	−0.02970 (5)	0.0547 (4)	
Cu29	0.29722 (5)	0.37054 (8)	−0.03934 (5)	0.0725 (5)	
Cu30	0.37891 (4)	0.28444 (5)	−0.00011 (5)	0.0526 (4)	
Cu31	0.29053 (5)	0.19625 (5)	0.07819 (5)	0.0558 (4)	
Cu32	0.21772 (5)	0.13500 (6)	0.01025 (5)	0.0584 (4)	
Cl1	0.21146 (14)	0.37186 (13)	0.59869 (11)	0.0872 (11)	
Cl2	0.17357 (9)	0.26957 (10)	0.49121 (10)	0.0506 (7)	
Cl3	0.30254 (9)	0.33463 (13)	0.51603 (12)	0.0676 (8)	
Cl4	0.25123 (12)	0.65910 (13)	0.55372 (10)	0.0698 (9)	
Cl5	0.16698 (12)	0.54014 (15)	0.59363 (11)	0.0775 (9)	
Cl6	0.24411 (10)	0.71154 (11)	0.41794 (10)	0.0596 (7)	
Cl7	0.05734 (10)	0.56982 (13)	0.52772 (12)	0.0727 (9)	
Cl8	0.34112 (11)	0.60748 (13)	0.47090 (12)	0.0725 (9)	
Cl9	0.03342 (11)	0.60746 (15)	0.39359 (13)	0.0813 (10)	
Cl10	0.01797 (10)	0.44353 (12)	0.42994 (12)	0.0674 (8)	
Cl11	0.09607 (10)	0.32954 (13)	0.39005 (11)	0.0674 (8)	
Cl12	0.19252 (11)	0.52940 (12)	0.26019 (10)	0.0651 (8)	
Cl13	0.14881 (11)	0.37007 (14)	0.28865 (10)	0.0691 (8)	
Cl14	0.28293 (10)	0.40796 (12)	0.31312 (9)	0.0584 (7)	
Cl15	0.34099 (11)	0.44163 (14)	0.43265 (11)	0.0729 (8)	
Cl16	0.14480 (14)	0.65016 (15)	0.33655 (12)	0.0871 (11)	
S1_1	0.1561 (9)	0.6131 (9)	0.4672 (12)	0.052 (3)	0.658 (9)
O1_1	0.0759 (5)	0.8191 (6)	0.4557 (6)	0.113 (5)	0.658 (9)
O2_1	0.0863 (8)	0.7659 (9)	0.5246 (7)	0.118 (5)	0.658 (9)
N1_1	0.1252 (7)	0.7406 (8)	0.4156 (6)	0.111 (6)	0.658 (9)
H1A_1	0.151161	0.719806	0.412019	0.166*	0.658 (9)
H1B_1	0.131609	0.780828	0.417792	0.166*	0.658 (9)
H1C_1	0.103787	0.733853	0.390538	0.166*	0.658 (9)
C1_1	0.1448 (12)	0.6878 (13)	0.4909 (10)	0.070 (4)	0.658 (9)
H1D_1	0.136453	0.683168	0.522750	0.084*	0.658 (9)
H1E_1	0.172790	0.712814	0.492789	0.084*	0.658 (9)
C2_1	0.1065 (7)	0.7191 (7)	0.4607 (6)	0.080 (4)	0.658 (9)
H2_1	0.081462	0.689175	0.451812	0.096*	0.658 (9)
C3_1	0.0861 (7)	0.7744 (8)	0.4811 (7)	0.090 (4)	0.658 (9)
S1A_1	0.1520 (18)	0.6071 (19)	0.464 (2)	0.059 (5)	0.342 (9)
O1A_1	0.0945 (11)	0.8049 (13)	0.5349 (12)	0.105 (7)	0.342 (9)
O2A_1	0.1397 (11)	0.7542 (13)	0.5775 (12)	0.101 (8)	0.342 (9)
N1A_1	0.0682 (12)	0.7037 (16)	0.4767 (13)	0.103 (7)	0.342 (9)
H1AA_1	0.061418	0.664940	0.467933	0.154*	0.342 (9)
H1AB_1	0.071144	0.726452	0.451103	0.154*	0.342 (9)
H1AC_1	0.045293	0.718996	0.491569	0.154*	0.342 (9)
C1A_1	0.140 (2)	0.685 (3)	0.483 (2)	0.070 (4)	0.342 (9)
H1BA_1	0.169334	0.700592	0.498029	0.084*	0.342 (9)
H1BB_1	0.132959	0.708072	0.453391	0.084*	0.342 (9)
C2A_1	0.1108 (13)	0.7049 (16)	0.5080 (15)	0.080 (4)	0.342 (9)
H2A_1	0.107182	0.669949	0.529161	0.096*	0.342 (9)
C3A_1	0.1142 (19)	0.759 (2)	0.542 (2)	0.089 (5)	0.342 (9)
S1_2	0.26016 (10)	0.49555 (12)	0.51876 (9)	0.0543 (7)	
O1_2	0.3397 (6)	0.4979 (8)	0.6846 (5)	0.194 (7)	
O2_2	0.3717 (6)	0.4512 (8)	0.6279 (5)	0.207 (7)	
N1_2	0.2702 (5)	0.5443 (6)	0.6317 (5)	0.134 (5)	
H1A_2	0.283616	0.579645	0.624647	0.202*	
H1B_2	0.271827	0.540574	0.663135	0.202*	
H1C_2	0.240393	0.544265	0.618745	0.202*	
C1_2	0.3100 (4)	0.4995 (7)	0.5638 (4)	0.087 (3)	
H1AA_2	0.332332	0.467894	0.557937	0.104*	
H1AB_2	0.325032	0.539330	0.562332	0.104*	
C2_2	0.2956 (5)	0.4905 (7)	0.6119 (4)	0.113 (4)	
H2_2	0.274803	0.454690	0.610315	0.136*	
C3_2	0.3374 (6)	0.4757 (9)	0.6452 (5)	0.139 (5)	
S1_3	0.13160 (9)	0.43683 (12)	0.49842 (10)	0.0559 (8)	
O1_3	0.0699 (3)	0.3400 (3)	0.6162 (3)	0.064 (2)	
O2_3	0.0193 (3)	0.2967 (3)	0.5619 (3)	0.062 (2)	
N1_3	0.0668 (3)	0.3145 (4)	0.4932 (3)	0.060 (2)	
H1A_3	0.052131	0.343936	0.475120	0.091*	
H1B_3	0.046652	0.284637	0.497854	0.091*	
H1C_3	0.089799	0.298864	0.478603	0.091*	
C1_3	0.0914 (4)	0.4085 (4)	0.5362 (4)	0.058 (3)	
H1D_3	0.100998	0.423664	0.568113	0.069*	
H1DE_3	0.060896	0.425945	0.525503	0.069*	
C2_3	0.0863 (3)	0.3404 (4)	0.5387 (3)	0.054 (3)	
H2_3	0.117107	0.322164	0.548152	0.064*	
C3_3	0.0555 (3)	0.3246 (4)	0.5750 (4)	0.053 (3)	
S1_4	0.24826 (10)	0.55195 (11)	0.38094 (10)	0.0555 (8)	
O1_4	0.3746 (4)	0.5903 (4)	0.2768 (4)	0.099 (3)	
O2_4	0.3076 (4)	0.6387 (5)	0.2617 (3)	0.100 (3)	
N1_4	0.3531 (4)	0.5391 (5)	0.3535 (3)	0.086 (3)	
H1A_4	0.365353	0.506414	0.340640	0.128*	
H1B_4	0.342295	0.527912	0.380334	0.128*	
H1C_4	0.375056	0.567934	0.360014	0.128*	
C1_4	0.2815 (4)	0.5998 (4)	0.3464 (4)	0.070 (4)	
H1AA_4	0.260230	0.623059	0.323806	0.085*	
H1AB_4	0.299331	0.629054	0.367322	0.085*	
C2_4	0.3144 (4)	0.5642 (5)	0.3198 (4)	0.076 (4)	
H2_4	0.297116	0.529201	0.304379	0.091*	
C3_4	0.3353 (5)	0.5980 (6)	0.2830 (5)	0.085 (4)	
S1_5	0.2212 (13)	0.3822 (12)	0.4101 (14)	0.042 (3)	0.549 (19)
O1_5	0.3101 (6)	0.2390 (8)	0.3591 (7)	0.065 (4)	0.549 (19)
O2_5	0.2538 (7)	0.1796 (7)	0.3275 (7)	0.070 (4)	0.549 (19)
N1_5	0.1852 (7)	0.2529 (8)	0.3495 (9)	0.070 (5)	0.549 (19)
H1A_5	0.179836	0.233180	0.375827	0.105*	0.549 (19)
H1B_5	0.181330	0.226863	0.324919	0.105*	0.549 (19)
H1C_5	0.165145	0.284184	0.343973	0.105*	0.549 (19)
C1_5	0.2459 (11)	0.3084 (13)	0.4011 (10)	0.048 (4)	0.549 (19)
H1AA_5	0.279575	0.312745	0.406670	0.058*	0.549 (19)
H1AB_5	0.237115	0.281074	0.425576	0.058*	0.549 (19)
C2_5	0.2343 (7)	0.2773 (8)	0.3556 (7)	0.053 (3)	0.549 (19)
H2_5	0.236155	0.308172	0.330686	0.063*	0.549 (19)
C3_5	0.2687 (7)	0.2277 (8)	0.3484 (9)	0.056 (4)	0.549 (19)
S1A_5	0.2191 (17)	0.3740 (15)	0.4142 (18)	0.047 (4)	0.451 (19)
O1A_5	0.2899 (9)	0.2343 (11)	0.3394 (8)	0.062 (5)	0.451 (19)
O2A_5	0.2275 (9)	0.1722 (9)	0.3300 (8)	0.067 (5)	0.451 (19)
N1A_5	0.1740 (8)	0.2498 (11)	0.3727 (9)	0.065 (5)	0.451 (19)
H1A1_5	0.150234	0.250176	0.349289	0.097*	0.451 (19)
H1A2_5	0.167656	0.274871	0.395968	0.097*	0.451 (19)
H1A3_5	0.178272	0.211565	0.384006	0.097*	0.451 (19)
C1A_5	0.2514 (16)	0.3002 (19)	0.4055 (15)	0.055 (4)	0.451 (19)
H1AC_5	0.282546	0.307891	0.397360	0.066*	0.451 (19)
H1AD_5	0.253385	0.273703	0.433267	0.066*	0.451 (19)
C2A_5	0.2129 (12)	0.2691 (12)	0.3560 (11)	0.058 (4)	0.451 (19)
H2A_5	0.205748	0.299946	0.330870	0.070*	0.451 (19)
C3A_5	0.2450 (11)	0.2183 (12)	0.3405 (11)	0.059 (4)	0.451 (19)
S1_6	0.11738 (19)	0.49435 (19)	0.36352 (19)	0.0583 (11)	0.826 (8)
O1_6	0.0079 (5)	0.5202 (8)	0.2134 (5)	0.140 (5)	0.826 (8)
O2_6	0.0236 (6)	0.4271 (7)	0.2359 (6)	0.157 (6)	0.826 (8)
N1_6	0.0591 (6)	0.5742 (6)	0.2781 (6)	0.118 (5)	0.826 (8)
H1A_6	0.028620	0.579676	0.279043	0.176*	0.826 (8)
H1B_6	0.067484	0.593466	0.252741	0.176*	0.826 (8)
H1C_6	0.075139	0.589663	0.304429	0.176*	0.826 (8)
C1_6	0.0642 (5)	0.4796 (8)	0.3219 (5)	0.092 (3)	0.826 (8)
H1D_6	0.058696	0.435578	0.319164	0.110*	0.826 (8)
H1E_6	0.037551	0.498417	0.333876	0.110*	0.826 (8)
C2_6	0.0694 (6)	0.5054 (7)	0.2748 (5)	0.109 (4)	0.826 (8)
H2_6	0.100328	0.497319	0.265367	0.131*	0.826 (8)
C3_6	0.0289 (7)	0.4819 (6)	0.2374 (7)	0.125 (5)	0.826 (8)
O3_6	0.0965 (9)	0.5789 (13)	0.1979 (8)	0.174 (11)	0.63 (3)
O4_6	0.1142 (9)	0.4710 (12)	0.1799 (9)	0.126 (15)	0.34 (2)
H4A_6	0.104 (3)	0.457 (2)	0.2032 (15)	0.189*	0.34 (2)
H4B_6	0.119 (4)	0.5077 (14)	0.186 (3)	0.189*	0.34 (2)
S1A_6	0.1183 (9)	0.4940 (7)	0.3643 (9)	0.076 (5)	0.174 (8)
O1A_6	−0.0009 (18)	0.566 (3)	0.253 (2)	0.131 (9)	0.174 (8)
O2A_6	0.0670 (19)	0.557 (3)	0.230 (2)	0.131 (7)	0.174 (8)
N1A_6	0.039 (2)	0.421 (2)	0.298 (3)	0.115 (9)	0.174 (8)
H1AA_6	0.046971	0.399268	0.273729	0.172*	0.174 (8)
H1AB_6	0.008531	0.416314	0.299682	0.172*	0.174 (8)
H1AC_6	0.055404	0.408946	0.324933	0.172*	0.174 (8)
C1A_6	0.1033 (14)	0.491 (3)	0.2993 (12)	0.093 (4)	0.174 (8)
H1BA_6	0.115553	0.526535	0.284486	0.111*	0.174 (8)
H1BB_6	0.116165	0.453884	0.286595	0.111*	0.174 (8)
C2A_6	0.049 (2)	0.490 (2)	0.290 (2)	0.108 (4)	0.174 (8)
H2A_6	0.040481	0.508481	0.318976	0.130*	0.174 (8)
C3A_6	0.0366 (16)	0.543 (2)	0.2528 (15)	0.118 (5)	0.174 (8)
S1_7	0.36689 (8)	0.38424 (10)	0.00077 (8)	0.0404 (6)	
O1_7	0.4468 (2)	0.4789 (3)	−0.1283 (3)	0.0589 (19)	
O2_7	0.4529 (3)	0.5238 (3)	−0.0570 (3)	0.071 (2)	
N1_7	0.3819 (3)	0.4029 (4)	−0.1109 (3)	0.060 (2)	
H1A_7	0.378061	0.421294	−0.139249	0.091*	
H1B_7	0.354802	0.386784	−0.105029	0.091*	
H1C_7	0.403111	0.373007	−0.110793	0.091*	
C1_7	0.4124 (3)	0.4198 (4)	−0.0277 (3)	0.043 (2)	
H1AA_7	0.427358	0.451126	−0.006499	0.051*	
H1AB_7	0.435787	0.388758	−0.031710	0.051*	
C2_7	0.3979 (4)	0.4484 (4)	−0.0740 (3)	0.056 (3)	
H2_7	0.371636	0.475948	−0.070501	0.067*	
C3_7	0.4356 (4)	0.4859 (4)	−0.0895 (4)	0.058 (3)	
S1_8	0.23844 (8)	0.32201 (10)	−0.01282 (9)	0.0447 (6)	
O1_8	0.1102 (4)	0.2923 (6)	−0.1457 (4)	0.126 (4)	
O2_8	0.0919 (4)	0.2791 (7)	−0.0744 (4)	0.135 (5)	
N1_8	0.2039 (4)	0.2799 (7)	−0.1205 (4)	0.113 (4)	
H1A_8	0.195095	0.308718	−0.142253	0.170*	
H1B_8	0.202257	0.242869	−0.134323	0.170*	
H1C_8	0.233226	0.287100	−0.107569	0.170*	
C1_8	0.1848 (4)	0.3330 (5)	−0.0509 (4)	0.078 (4)	
H1AA_8	0.159872	0.339170	−0.031531	0.093*	
H1AB_8	0.187185	0.370161	−0.069566	0.093*	
C2_8	0.1727 (4)	0.2815 (6)	−0.0831 (4)	0.084 (4)	
H2_8	0.177643	0.243402	−0.064519	0.101*	
C3_8	0.1220 (4)	0.2840 (8)	−0.1037 (5)	0.097 (5)	
S1_9	0.28393 (9)	0.43517 (10)	0.09544 (9)	0.0456 (6)	
O1_9	0.2567 (3)	0.6072 (3)	0.1752 (3)	0.067 (2)	
O2_9	0.1845 (3)	0.6009 (3)	0.1452 (3)	0.066 (2)	
N1_9	0.1864 (3)	0.4857 (3)	0.1237 (3)	0.062 (3)	
H1A_9	0.177384	0.501291	0.094865	0.093*	
H1B_9	0.164774	0.493925	0.142642	0.093*	
H1C_9	0.189821	0.444984	0.121499	0.093*	
C1_9	0.2675 (4)	0.5114 (4)	0.1116 (3)	0.050 (3)	
H1D_9	0.295158	0.532541	0.126530	0.060*	
H1DE_9	0.256105	0.533730	0.082830	0.060*	
C2_9	0.2317 (3)	0.5140 (4)	0.1439 (3)	0.053 (3)	
H2_9	0.243468	0.491512	0.172971	0.064*	
C3_9	0.2233 (4)	0.5788 (4)	0.1571 (4)	0.057 (3)	
S1_10	0.34156 (9)	0.20907 (10)	0.02913 (9)	0.0453 (6)	
O1_10	0.3877 (2)	0.0336 (3)	−0.0650 (3)	0.0577 (19)	
O2_10	0.4188 (2)	0.0312 (3)	0.0089 (3)	0.065 (2)	
N1_10	0.3713 (3)	0.1500 (3)	−0.0661 (3)	0.056 (2)	
H1A_10	0.392315	0.145372	−0.086286	0.083*	
H1B_10	0.365034	0.189931	−0.063085	0.083*	
H1C_10	0.345224	0.129913	−0.077095	0.083*	
C1_10	0.3564 (3)	0.1313 (4)	0.0164 (4)	0.049 (3)	
H1D_10	0.369984	0.111794	0.045775	0.059*	
H1DE_10	0.327957	0.109160	0.005012	0.059*	
C2_10	0.3901 (3)	0.1250 (4)	−0.0198 (3)	0.050 (2)	
H2_10	0.419376	0.145957	−0.008207	0.060*	
C3_10	0.3996 (4)	0.0576 (4)	−0.0274 (4)	0.060 (3)	
S1_11	0.38783 (9)	0.32509 (11)	0.13855 (9)	0.0448 (6)	
O1_11	0.5256 (3)	0.3758 (6)	0.2567 (3)	0.105 (3)	
O2_11	0.4636 (4)	0.3444 (6)	0.2834 (3)	0.129 (4)	
N1_11	0.4846 (3)	0.4092 (4)	0.1750 (3)	0.065 (2)	
H1A_11	0.508989	0.386110	0.169671	0.097*	
H1B_11	0.494809	0.444097	0.189389	0.097*	
H1C_11	0.467496	0.418043	0.147344	0.097*	
C1_11	0.4369 (3)	0.3176 (5)	0.1838 (4)	0.060 (3)	
H1AA_11	0.427724	0.291727	0.208978	0.072*	
H1AB_11	0.461595	0.296187	0.170304	0.072*	
C2_11	0.4561 (3)	0.3754 (5)	0.2053 (3)	0.064 (3)	
H2_11	0.430147	0.401846	0.211697	0.077*	
C3_11	0.4854 (4)	0.3628 (6)	0.2501 (3)	0.079 (4)	
S1_12	0.25882 (9)	0.25913 (10)	0.12410 (9)	0.0461 (6)	
O1_12	0.1909 (3)	0.1233 (4)	0.2376 (3)	0.084 (3)	
O2_12	0.1752 (3)	0.2212 (4)	0.2429 (3)	0.087 (3)	
N1_12	0.2607 (3)	0.1363 (4)	0.1905 (3)	0.066 (3)	
H1A_12	0.241493	0.111217	0.172395	0.099*	
H1B_12	0.271226	0.117414	0.217684	0.099*	
H1C_12	0.284796	0.146215	0.175143	0.099*	
C1_12	0.2162 (3)	0.2244 (4)	0.1561 (3)	0.053 (3)	
H1AA_12	0.194705	0.256091	0.163770	0.064*	
H1AB_12	0.198493	0.194725	0.135472	0.064*	
C2_12	0.2355 (3)	0.1927 (4)	0.2007 (3)	0.054 (3)	
H2_12	0.257345	0.220635	0.219442	0.065*	
C3_12	0.1972 (4)	0.1767 (5)	0.2292 (4)	0.075 (4)	
O1_13	0.4453 (3)	0.3261 (4)	−0.1455 (3)	0.075 (2)	
H1A_13	0.4610 (11)	0.302 (2)	−0.1292 (14)	0.113*	
H1B_13	0.4318 (11)	0.310 (2)	−0.1691 (11)	0.113*	

### Atomic displacement parameters (Å^2^)

**Table:**

	*U*^11^	*U*^22^	*U*^33^	*U*^12^	*U*^13^	*U*^23^
Cu1	0.0695 (9)	0.0469 (8)	0.0612 (9)	0.0022 (6)	0.0228 (7)	0.0054 (6)
Cu2	0.0668 (10)	0.0784 (10)	0.0671 (10)	−0.0047 (8)	0.0277 (7)	0.0144 (8)
Cu3	0.0886 (11)	0.0427 (8)	0.0688 (10)	0.0137 (7)	0.0043 (8)	−0.0085 (7)
Cu4	0.0662 (8)	0.0386 (7)	0.0706 (9)	0.0057 (6)	0.0381 (7)	0.0037 (6)
Cu5	0.0663 (9)	0.0384 (7)	0.0702 (9)	−0.0073 (6)	0.0271 (7)	−0.0109 (6)
Cu6	0.0628 (9)	0.0400 (7)	0.0946 (11)	0.0042 (6)	0.0422 (8)	0.0084 (7)
Cu7	0.0564 (9)	0.0954 (13)	0.0946 (12)	0.0039 (8)	0.0363 (8)	0.0186 (10)
Cu8	0.0959 (13)	0.0811 (12)	0.1121 (15)	0.0341 (10)	−0.0019 (11)	−0.0430 (11)
Cu9	0.0705 (10)	0.0455 (8)	0.1142 (13)	0.0015 (7)	0.0377 (9)	0.0014 (8)
Cu10	0.0987 (11)	0.0370 (7)	0.0857 (11)	−0.0071 (7)	0.0573 (9)	−0.0131 (7)
Cu11	0.0792 (10)	0.0467 (8)	0.0690 (10)	−0.0138 (6)	0.0373 (8)	−0.0074 (7)
Cu12	0.0805 (10)	0.0476 (8)	0.0625 (9)	−0.0060 (7)	0.0340 (7)	0.0006 (6)
Cu13	0.0888 (11)	0.0962 (12)	0.0579 (10)	0.0041 (9)	0.0378 (8)	0.0132 (8)
Cu14	0.0789 (10)	0.0568 (9)	0.0713 (10)	0.0205 (7)	0.0040 (8)	−0.0159 (7)
Cu15	0.1385 (15)	0.0323 (7)	0.0943 (12)	−0.0080 (8)	0.0829 (11)	−0.0085 (7)
Cu16	0.0938 (11)	0.0477 (8)	0.0590 (9)	0.0080 (7)	0.0299 (8)	0.0013 (6)
Cl17	0.0777 (17)	0.0387 (12)	0.0531 (15)	0.0059 (12)	0.0316 (13)	−0.0027 (11)
Cl18	0.094 (2)	0.0466 (14)	0.0667 (18)	−0.0101 (14)	0.0416 (16)	−0.0090 (13)
Cl19	0.0714 (17)	0.0458 (13)	0.0589 (16)	0.0051 (12)	0.0274 (13)	0.0043 (12)
Cl20	0.0591 (15)	0.0337 (12)	0.0680 (17)	0.0063 (10)	0.0297 (13)	0.0058 (11)
Cl21	0.0609 (16)	0.0498 (14)	0.0589 (16)	0.0025 (12)	0.0236 (13)	0.0037 (12)
Cl22	0.0670 (16)	0.0292 (11)	0.0604 (16)	0.0047 (11)	0.0278 (13)	0.0054 (11)
Cl23	0.0636 (16)	0.0382 (12)	0.0539 (15)	0.0063 (11)	0.0185 (12)	0.0020 (11)
Cl24	0.0442 (13)	0.0399 (12)	0.0617 (15)	0.0011 (10)	0.0193 (11)	−0.0059 (11)
Cl25	0.0465 (14)	0.0349 (11)	0.0600 (15)	−0.0022 (9)	0.0231 (11)	−0.0013 (10)
Cl26	0.0774 (18)	0.0352 (12)	0.0620 (16)	−0.0057 (12)	0.0242 (14)	−0.0025 (11)
Cl27	0.0633 (17)	0.0524 (15)	0.081 (2)	0.0136 (13)	0.0367 (15)	0.0168 (14)
Cl28	0.087 (2)	0.0369 (13)	0.089 (2)	0.0048 (13)	0.0405 (17)	−0.0066 (13)
Cl29	0.0744 (19)	0.0572 (17)	0.094 (2)	0.0090 (14)	0.0406 (17)	0.0264 (16)
Cl30	0.0598 (16)	0.0381 (13)	0.0799 (19)	−0.0079 (11)	0.0280 (14)	−0.0121 (13)
Cl31	0.0727 (17)	0.0331 (12)	0.0631 (17)	−0.0138 (11)	0.0298 (14)	−0.0096 (11)
Cl32	0.0692 (17)	0.0268 (11)	0.0597 (16)	−0.0071 (10)	0.0311 (13)	−0.0057 (10)
Cu17	0.0797 (9)	0.0432 (7)	0.0547 (8)	0.0008 (6)	0.0280 (7)	−0.0016 (6)
Cu18	0.0648 (9)	0.0380 (7)	0.0666 (9)	0.0070 (6)	0.0212 (7)	−0.0037 (6)
Cu19	0.0596 (8)	0.0526 (8)	0.0557 (8)	−0.0054 (6)	0.0251 (6)	0.0032 (6)
Cu20	0.0690 (8)	0.0270 (6)	0.0577 (8)	0.0020 (5)	0.0327 (6)	0.0005 (5)
Cu21	0.0868 (10)	0.0319 (7)	0.0704 (9)	0.0149 (6)	0.0484 (8)	0.0098 (6)
Cu22	0.0513 (7)	0.0387 (7)	0.0482 (7)	−0.0001 (5)	0.0196 (6)	0.0019 (5)
Cu23	0.0639 (9)	0.0450 (7)	0.0707 (9)	0.0076 (6)	0.0214 (7)	−0.0111 (6)
Cu24	0.0809 (11)	0.0362 (7)	0.0980 (12)	0.0146 (7)	0.0008 (9)	−0.0216 (7)
Cu25	0.0714 (9)	0.0493 (8)	0.0604 (9)	−0.0164 (7)	0.0228 (7)	0.0064 (6)
Cu26	0.0529 (8)	0.0410 (7)	0.0692 (9)	0.0003 (6)	0.0230 (6)	−0.0047 (6)
Cu27	0.0649 (8)	0.0309 (6)	0.0664 (9)	0.0079 (5)	0.0196 (7)	−0.0045 (6)
Cu28	0.0643 (9)	0.0385 (7)	0.0673 (9)	0.0042 (6)	0.0313 (7)	0.0044 (6)
Cu29	0.0535 (9)	0.0941 (12)	0.0710 (10)	−0.0127 (8)	0.0128 (7)	0.0350 (9)
Cu30	0.0597 (8)	0.0277 (6)	0.0773 (9)	−0.0017 (5)	0.0358 (7)	−0.0006 (6)
Cu31	0.0714 (9)	0.0310 (7)	0.0737 (9)	−0.0083 (6)	0.0424 (7)	−0.0090 (6)
Cu32	0.0748 (9)	0.0384 (7)	0.0691 (9)	−0.0047 (6)	0.0367 (7)	−0.0057 (6)
Cl1	0.157 (3)	0.0459 (16)	0.0664 (19)	0.0100 (18)	0.044 (2)	0.0021 (14)
Cl2	0.0529 (14)	0.0330 (12)	0.0703 (17)	0.0034 (10)	0.0246 (13)	0.0023 (11)
Cl3	0.0546 (16)	0.0535 (16)	0.096 (2)	0.0047 (12)	0.0133 (15)	0.0251 (15)
Cl4	0.105 (2)	0.0479 (15)	0.0650 (18)	−0.0293 (15)	0.0416 (17)	−0.0163 (13)
Cl5	0.099 (2)	0.0708 (19)	0.0665 (19)	−0.0157 (17)	0.0264 (17)	−0.0030 (16)
Cl6	0.087 (2)	0.0333 (12)	0.0648 (17)	−0.0048 (12)	0.0320 (15)	−0.0064 (12)
Cl7	0.0625 (18)	0.0525 (16)	0.111 (3)	−0.0026 (13)	0.0429 (17)	−0.0128 (16)
Cl8	0.076 (2)	0.0488 (16)	0.099 (2)	−0.0061 (14)	0.0352 (17)	−0.0013 (15)
Cl9	0.076 (2)	0.0588 (18)	0.114 (3)	0.0073 (15)	0.0324 (19)	0.0191 (17)
Cl10	0.0578 (16)	0.0471 (15)	0.105 (2)	−0.0066 (12)	0.0384 (16)	−0.0053 (15)
Cl11	0.0685 (19)	0.0560 (16)	0.079 (2)	−0.0113 (14)	0.0156 (16)	0.0024 (14)
Cl12	0.100 (2)	0.0477 (14)	0.0542 (16)	0.0138 (14)	0.0366 (15)	0.0073 (12)
Cl13	0.090 (2)	0.0618 (17)	0.0603 (17)	−0.0062 (15)	0.0264 (15)	−0.0095 (14)
Cl14	0.0828 (19)	0.0454 (13)	0.0542 (15)	0.0034 (12)	0.0363 (14)	−0.0042 (11)
Cl15	0.077 (2)	0.0669 (18)	0.080 (2)	0.0005 (15)	0.0287 (16)	0.0111 (16)
Cl16	0.132 (3)	0.0600 (19)	0.070 (2)	0.0096 (19)	0.014 (2)	0.0117 (16)
S1_1	0.066 (5)	0.026 (4)	0.073 (5)	−0.008 (3)	0.039 (4)	−0.008 (3)
O1_1	0.106 (11)	0.058 (8)	0.170 (13)	0.028 (8)	−0.003 (10)	−0.017 (8)
O2_1	0.147 (11)	0.083 (9)	0.129 (9)	0.013 (10)	0.042 (10)	−0.026 (9)
N1_1	0.152 (15)	0.086 (12)	0.099 (11)	0.053 (11)	0.036 (10)	−0.006 (9)
C1_1	0.084 (7)	0.042 (6)	0.090 (8)	0.003 (6)	0.034 (6)	−0.014 (6)
C2_1	0.089 (8)	0.050 (6)	0.104 (8)	0.014 (6)	0.026 (7)	−0.022 (6)
C3_1	0.097 (8)	0.055 (6)	0.119 (8)	0.017 (6)	0.026 (7)	−0.031 (7)
S1A_1	0.071 (8)	0.036 (8)	0.075 (7)	0.005 (8)	0.032 (7)	−0.006 (8)
O1A_1	0.124 (14)	0.059 (11)	0.137 (15)	0.017 (11)	0.034 (13)	−0.039 (11)
O2A_1	0.111 (17)	0.069 (15)	0.123 (15)	−0.002 (13)	0.018 (12)	−0.021 (13)
N1A_1	0.107 (11)	0.072 (12)	0.127 (14)	0.008 (12)	0.006 (11)	−0.018 (12)
C1A_1	0.085 (7)	0.040 (7)	0.089 (8)	0.003 (7)	0.030 (7)	−0.015 (7)
C2A_1	0.089 (8)	0.050 (7)	0.105 (8)	0.014 (7)	0.028 (7)	−0.024 (7)
C3A_1	0.097 (11)	0.060 (8)	0.114 (10)	0.014 (9)	0.028 (9)	−0.031 (8)
S1_2	0.0725 (18)	0.0388 (13)	0.0534 (16)	−0.0094 (12)	0.0153 (14)	0.0002 (12)
O1_2	0.244 (16)	0.191 (14)	0.131 (10)	0.066 (12)	−0.030 (11)	−0.056 (10)
O2_2	0.272 (16)	0.206 (15)	0.129 (11)	0.112 (13)	−0.026 (11)	−0.051 (11)
N1_2	0.154 (12)	0.099 (10)	0.150 (12)	0.013 (9)	0.018 (10)	−0.018 (9)
C1_2	0.106 (8)	0.069 (7)	0.082 (7)	−0.001 (6)	−0.001 (6)	0.000 (6)
C2_2	0.157 (10)	0.088 (8)	0.088 (7)	0.021 (7)	−0.009 (7)	−0.002 (7)
C3_2	0.187 (12)	0.119 (10)	0.097 (9)	0.049 (9)	−0.040 (9)	−0.019 (8)
S1_3	0.0564 (16)	0.0403 (14)	0.0796 (19)	−0.0027 (11)	0.0417 (15)	−0.0025 (13)
O1_3	0.069 (5)	0.059 (5)	0.069 (5)	−0.006 (4)	0.028 (4)	0.005 (4)
O2_3	0.074 (5)	0.047 (4)	0.073 (5)	−0.013 (4)	0.037 (4)	0.007 (4)
N1_3	0.060 (5)	0.036 (4)	0.093 (7)	−0.008 (4)	0.037 (5)	−0.010 (4)
C1_3	0.056 (6)	0.051 (6)	0.075 (7)	0.003 (5)	0.043 (6)	0.002 (5)
C2_3	0.060 (6)	0.029 (5)	0.080 (8)	−0.002 (4)	0.038 (6)	−0.005 (5)
C3_3	0.045 (6)	0.041 (6)	0.082 (9)	0.003 (5)	0.034 (6)	0.008 (5)
S1_4	0.085 (2)	0.0317 (13)	0.0572 (16)	−0.0029 (12)	0.0379 (15)	−0.0028 (11)
O1_4	0.134 (9)	0.056 (5)	0.121 (8)	−0.012 (5)	0.074 (7)	0.008 (5)
O2_4	0.142 (9)	0.088 (7)	0.074 (6)	−0.012 (7)	0.033 (6)	0.016 (5)
N1_4	0.104 (8)	0.084 (8)	0.078 (7)	−0.006 (6)	0.048 (7)	0.018 (6)
C1_4	0.114 (10)	0.037 (6)	0.070 (8)	−0.016 (6)	0.048 (7)	0.000 (5)
C2_4	0.102 (10)	0.047 (6)	0.088 (9)	−0.008 (6)	0.048 (8)	0.003 (6)
C3_4	0.105 (11)	0.073 (9)	0.090 (10)	−0.009 (8)	0.058 (9)	0.017 (8)
S1_5	0.053 (4)	0.023 (6)	0.056 (7)	−0.001 (4)	0.027 (4)	0.005 (5)
O1_5	0.081 (9)	0.044 (7)	0.074 (11)	0.009 (7)	0.027 (8)	−0.002 (8)
O2_5	0.095 (12)	0.046 (7)	0.073 (9)	0.009 (8)	0.023 (9)	−0.019 (6)
N1_5	0.077 (9)	0.043 (8)	0.093 (12)	−0.009 (8)	0.026 (9)	−0.019 (9)
C1_5	0.067 (7)	0.020 (7)	0.064 (7)	0.008 (6)	0.031 (6)	0.000 (6)
C2_5	0.072 (7)	0.029 (6)	0.063 (6)	0.007 (6)	0.032 (6)	0.000 (5)
C3_5	0.081 (9)	0.034 (6)	0.059 (7)	0.013 (7)	0.028 (8)	−0.006 (6)
S1A_5	0.064 (6)	0.025 (7)	0.059 (7)	0.004 (6)	0.031 (5)	0.006 (6)
O1A_5	0.077 (11)	0.055 (9)	0.058 (11)	0.011 (8)	0.025 (9)	−0.003 (9)
O2A_5	0.101 (13)	0.045 (7)	0.062 (9)	0.005 (8)	0.033 (11)	−0.016 (7)
N1A_5	0.084 (10)	0.059 (10)	0.059 (12)	0.001 (8)	0.039 (9)	−0.022 (9)
C1A_5	0.076 (8)	0.029 (8)	0.067 (7)	0.006 (7)	0.033 (7)	−0.003 (7)
C2A_5	0.079 (8)	0.034 (6)	0.067 (7)	0.011 (6)	0.031 (7)	−0.002 (6)
C3A_5	0.081 (9)	0.035 (6)	0.065 (7)	0.010 (7)	0.030 (7)	−0.004 (6)
S1_6	0.069 (2)	0.045 (2)	0.065 (2)	0.0061 (19)	0.019 (2)	0.001 (2)
O1_6	0.136 (10)	0.172 (11)	0.101 (9)	0.010 (9)	−0.027 (8)	−0.019 (8)
O2_6	0.150 (12)	0.146 (10)	0.161 (12)	−0.003 (10)	−0.035 (10)	−0.035 (10)
N1_6	0.125 (10)	0.112 (9)	0.110 (10)	−0.003 (8)	−0.007 (9)	−0.007 (8)
C1_6	0.098 (7)	0.086 (7)	0.088 (6)	0.010 (6)	0.000 (6)	−0.012 (6)
C2_6	0.113 (8)	0.112 (7)	0.097 (7)	0.001 (7)	−0.007 (6)	−0.007 (7)
C3_6	0.127 (9)	0.136 (8)	0.105 (8)	−0.001 (8)	−0.013 (7)	−0.015 (8)
O3_6	0.17 (2)	0.19 (2)	0.155 (19)	−0.008 (16)	0.015 (15)	0.008 (16)
O4_6	0.077 (18)	0.18 (3)	0.13 (2)	0.046 (17)	0.046 (16)	0.041 (19)
S1A_6	0.085 (9)	0.067 (9)	0.077 (9)	0.007 (9)	0.009 (9)	−0.003 (9)
O1A_6	0.127 (14)	0.144 (17)	0.117 (17)	0.011 (14)	−0.008 (14)	−0.016 (16)
O2A_6	0.137 (13)	0.150 (14)	0.102 (14)	−0.007 (13)	−0.003 (12)	0.007 (12)
N1A_6	0.112 (17)	0.114 (13)	0.114 (17)	−0.004 (14)	−0.002 (17)	−0.005 (16)
C1A_6	0.102 (8)	0.086 (8)	0.088 (8)	0.004 (7)	0.002 (7)	−0.007 (8)
C2A_6	0.113 (8)	0.110 (8)	0.098 (8)	0.001 (8)	−0.007 (8)	−0.012 (8)
C3A_6	0.120 (9)	0.125 (9)	0.102 (9)	0.001 (9)	−0.010 (8)	−0.007 (8)
S1_7	0.0493 (14)	0.0293 (11)	0.0467 (14)	0.0008 (9)	0.0215 (11)	0.0035 (10)
O1_7	0.066 (5)	0.049 (4)	0.065 (5)	0.000 (3)	0.020 (4)	0.012 (4)
O2_7	0.085 (6)	0.051 (5)	0.084 (6)	−0.011 (4)	0.037 (5)	−0.004 (4)
N1_7	0.070 (6)	0.063 (6)	0.051 (5)	−0.001 (5)	0.020 (5)	−0.001 (4)
C1_7	0.054 (6)	0.033 (5)	0.043 (5)	0.009 (4)	0.015 (4)	0.000 (4)
C2_7	0.080 (7)	0.032 (5)	0.063 (7)	0.001 (5)	0.036 (6)	0.000 (5)
C3_7	0.062 (7)	0.045 (6)	0.072 (8)	0.002 (5)	0.032 (6)	0.006 (6)
S1_8	0.0515 (15)	0.0308 (12)	0.0546 (16)	0.0010 (10)	0.0180 (12)	0.0011 (10)
O1_8	0.127 (9)	0.165 (12)	0.091 (8)	0.001 (8)	0.031 (7)	−0.010 (8)
O2_8	0.099 (8)	0.173 (13)	0.142 (10)	−0.001 (8)	0.054 (8)	−0.009 (9)
N1_8	0.096 (9)	0.126 (12)	0.121 (11)	0.001 (8)	0.027 (8)	−0.040 (9)
C1_8	0.093 (9)	0.066 (8)	0.073 (9)	−0.033 (7)	0.010 (7)	−0.001 (7)
C2_8	0.104 (11)	0.075 (9)	0.074 (9)	0.014 (8)	0.011 (8)	0.003 (7)
C3_8	0.083 (11)	0.119 (13)	0.091 (11)	0.004 (9)	0.016 (9)	−0.016 (10)
S1_9	0.0616 (16)	0.0257 (11)	0.0558 (15)	0.0067 (10)	0.0314 (13)	0.0023 (10)
O1_9	0.102 (6)	0.042 (4)	0.062 (5)	0.003 (4)	0.028 (5)	−0.010 (4)
O2_9	0.105 (6)	0.034 (4)	0.066 (5)	0.017 (4)	0.041 (5)	0.002 (3)
N1_9	0.081 (7)	0.034 (4)	0.079 (7)	0.022 (4)	0.038 (5)	0.007 (4)
C1_9	0.079 (7)	0.023 (4)	0.055 (6)	0.014 (4)	0.033 (5)	−0.001 (4)
C2_9	0.080 (8)	0.030 (5)	0.055 (6)	0.012 (5)	0.029 (6)	0.013 (4)
C3_9	0.092 (9)	0.028 (5)	0.057 (7)	0.014 (6)	0.035 (7)	−0.003 (5)
S1_10	0.0616 (16)	0.0238 (11)	0.0561 (15)	−0.0028 (10)	0.0292 (13)	−0.0019 (10)
O1_10	0.067 (5)	0.040 (4)	0.073 (5)	0.003 (3)	0.033 (4)	−0.007 (4)
O2_10	0.061 (5)	0.049 (4)	0.088 (6)	0.007 (3)	0.026 (4)	0.009 (4)
N1_10	0.076 (6)	0.037 (4)	0.060 (6)	0.007 (4)	0.034 (5)	−0.004 (4)
C1_10	0.065 (6)	0.031 (5)	0.059 (6)	−0.001 (4)	0.034 (5)	−0.005 (4)
C2_10	0.055 (6)	0.041 (5)	0.056 (6)	−0.003 (4)	0.021 (5)	−0.001 (5)
C3_10	0.070 (8)	0.045 (6)	0.073 (8)	0.001 (5)	0.045 (7)	−0.001 (6)
S1_11	0.0534 (15)	0.0369 (12)	0.0473 (15)	0.0039 (10)	0.0182 (12)	0.0020 (10)
O1_11	0.070 (6)	0.159 (10)	0.081 (7)	0.019 (7)	−0.015 (5)	0.000 (7)
O2_11	0.166 (11)	0.161 (11)	0.071 (7)	−0.008 (9)	0.053 (7)	0.008 (7)
N1_11	0.066 (6)	0.069 (6)	0.060 (6)	−0.009 (5)	0.010 (5)	−0.002 (5)
C1_11	0.066 (7)	0.055 (6)	0.063 (7)	0.001 (5)	0.024 (6)	0.001 (5)
C2_11	0.055 (7)	0.092 (9)	0.047 (6)	0.001 (6)	0.016 (5)	−0.005 (6)
C3_11	0.105 (11)	0.090 (10)	0.044 (7)	0.003 (8)	0.019 (7)	−0.001 (7)
S1_12	0.0602 (16)	0.0288 (12)	0.0551 (16)	−0.0066 (10)	0.0300 (13)	−0.0017 (10)
O1_12	0.118 (7)	0.061 (5)	0.083 (6)	−0.042 (5)	0.056 (5)	−0.008 (4)
O2_12	0.103 (7)	0.074 (6)	0.095 (7)	0.014 (5)	0.058 (6)	0.030 (5)
N1_12	0.086 (7)	0.056 (5)	0.062 (6)	0.010 (5)	0.038 (5)	0.010 (5)
C1_12	0.074 (7)	0.040 (5)	0.054 (6)	0.002 (5)	0.042 (6)	−0.004 (5)
C2_12	0.069 (7)	0.034 (5)	0.067 (7)	0.001 (5)	0.034 (6)	0.002 (5)
C3_12	0.083 (9)	0.074 (9)	0.074 (9)	−0.003 (7)	0.039 (7)	0.013 (7)
O1_13	0.091 (6)	0.064 (5)	0.069 (5)	−0.004 (4)	0.007 (5)	−0.009 (4)

### Geometric parameters (Å, º)

**Table:**

Cu1—Cl3	2.244 (3)	N1_1—H1B_1	0.9100
Cu1—Cl1	2.320 (3)	N1_1—H1C_1	0.9100
Cu1—Cl2	2.355 (3)	C1_1—C2_1	1.503 (19)
Cu1—Cl6^i^	2.572 (3)	C1_1—H1D_1	0.9900
Cu1—Cu4	2.900 (2)	C1_1—H1E_1	0.9900
Cu1—Cu2	3.048 (2)	C2_1—C3_1	1.518 (16)
Cu2—S1_2	2.243 (3)	C2_1—H2_1	1.0000
Cu2—S1_3	2.267 (3)	S1A_1—C1A_1	1.86 (5)
Cu2—Cl1	2.283 (3)	O1A_1—C3A_1	1.17 (6)
Cu2—Cu4	2.734 (2)	O2A_1—C3A_1	1.20 (6)
Cu2—Cu3	2.982 (2)	N1A_1—C2A_1	1.45 (5)
Cu2—Cu6	3.018 (2)	N1A_1—H1AA_1	0.9100
Cu3—S1_2	2.253 (3)	N1A_1—H1AB_1	0.9100
Cu3—S1_5	2.26 (4)	N1A_1—H1AC_1	0.9100
Cu3—S1A_5	2.28 (5)	C1A_1—C2A_1	1.26 (7)
Cu3—Cl3	2.299 (3)	C1A_1—H1BA_1	0.9900
Cu3—Cl15	2.673 (3)	C1A_1—H1BB_1	0.9900
Cu3—Cu12	2.890 (2)	C2A_1—C3A_1	1.54 (6)
Cu3—Cu15	2.914 (2)	C2A_1—H2A_1	1.0000
Cu3—Cu4	2.915 (2)	S1_2—C1_2	1.835 (11)
Cu4—S1A_5	2.14 (4)	O1_2—C3_2	1.235 (15)
Cu4—S1_3	2.223 (3)	O2_2—C3_2	1.302 (16)
Cu4—S1_5	2.30 (3)	N1_2—C2_2	1.553 (15)
Cu4—Cl2	2.318 (3)	N1_2—H1A_2	0.9100
Cu4—Cu7	2.976 (2)	N1_2—H1B_2	0.9100
Cu5—S1_1	2.21 (3)	N1_2—H1C_2	0.9100
Cu5—S1_2	2.232 (3)	C1_2—C2_2	1.520 (15)
Cu5—Cl4	2.288 (3)	C1_2—H1AA_2	0.9900
Cu5—S1A_1	2.31 (6)	C1_2—H1AB_2	0.9900
Cu5—Cu6	2.7449 (19)	C2_2—C3_2	1.499 (16)
Cu5—Cu12	2.7721 (18)	C2_2—H2_2	1.0000
Cu5—Cu10	2.830 (2)	S1_3—C1_3	1.824 (8)
Cu5—Cu11	2.8889 (18)	O1_3—C3_3	1.260 (11)
Cu6—S1A_1	2.21 (6)	O2_3—C3_3	1.248 (11)
Cu6—S1_1	2.29 (3)	N1_3—C2_3	1.482 (11)
Cu6—S1_3	2.298 (3)	N1_3—H1A_3	0.9100
Cu6—Cl7	2.363 (3)	N1_3—H1B_3	0.9100
Cu6—Cl5	2.435 (4)	N1_3—H1C_3	0.9100
Cu7—S1_3	2.230 (4)	C1_3—C2_3	1.517 (11)
Cu7—S1A_6	2.234 (17)	C1_3—H1D_3	0.9900
Cu7—S1_6	2.246 (5)	C1_3—H1DE_3	0.9900
Cu7—Cl10	2.328 (3)	C2_3—C3_3	1.513 (11)
Cu7—Cu14	2.855 (2)	C2_3—H2_3	1.0000
Cu7—Cu8	2.916 (3)	S1_4—C1_4	1.826 (9)
Cu8—S1A_1	2.10 (6)	O1_4—C3_4	1.208 (12)
Cu8—S1A_6	2.248 (18)	O2_4—C3_4	1.314 (13)
Cu8—S1_6	2.249 (5)	N1_4—C2_4	1.509 (13)
Cu8—Cl9	2.255 (4)	N1_4—H1A_4	0.9100
Cu8—S1_1	2.27 (3)	N1_4—H1B_4	0.9100
Cu8—Cu9	3.052 (3)	N1_4—H1C_4	0.9100
Cu9—Cl7	2.269 (4)	C1_4—C2_4	1.531 (13)
Cu9—Cl10	2.292 (3)	C1_4—H1AA_4	0.9900
Cu9—Cl9	2.369 (4)	C1_4—H1AB_4	0.9900
Cu9—Cl7^ii^	2.576 (3)	C2_4—C3_4	1.496 (13)
Cu10—S1_1	2.23 (3)	C2_4—H2_4	1.0000
Cu10—S1_4	2.236 (3)	S1_5—C1_5	1.819 (19)
Cu10—S1A_1	2.27 (6)	O1_5—C3_5	1.245 (17)
Cu10—Cl6	2.338 (3)	O2_5—C3_5	1.273 (17)
Cu10—Cu12	2.906 (2)	N1_5—C2_5	1.535 (17)
Cu10—Cu13	3.020 (2)	N1_5—H1A_5	0.9100
Cu10—Cu11	3.036 (2)	N1_5—H1B_5	0.9100
Cu11—Cl4	2.264 (3)	N1_5—H1C_5	0.9100
Cu11—Cl8	2.308 (3)	C1_5—C2_5	1.486 (19)
Cu11—Cl6	2.344 (3)	C1_5—H1AA_5	0.9900
Cu11—Cl2^iii^	2.593 (3)	C1_5—H1AB_5	0.9900
Cu11—Cu12	3.0223 (19)	C2_5—C3_5	1.525 (17)
Cu12—S1_4	2.251 (3)	C2_5—H2_5	1.0000
Cu12—S1_2	2.263 (3)	S1A_5—C1A_5	1.92 (3)
Cu12—Cl8	2.297 (3)	O1A_5—C3A_5	1.37 (3)
Cu12—Cu15	3.009 (2)	O2A_5—C3A_5	1.16 (3)
Cu13—S1A_6	2.228 (18)	N1A_5—C2A_5	1.37 (4)
Cu13—S1_6	2.237 (5)	N1A_5—H1A1_5	0.9100
Cu13—S1_4	2.293 (4)	N1A_5—H1A2_5	0.9100
Cu13—Cl12	2.300 (3)	N1A_5—H1A3_5	0.9100
Cu13—C1A_6	2.54 (4)	C1A_5—C2A_5	1.84 (5)
Cu13—Cu14	2.966 (2)	C1A_5—H1AC_5	0.9900
Cu13—Cu15	2.981 (3)	C1A_5—H1AD_5	0.9900
Cu13—Cu16	3.048 (2)	C2A_5—C3A_5	1.57 (3)
Cu14—S1_5	2.22 (4)	C2A_5—H2A_5	1.0000
Cu14—S1A_6	2.255 (18)	S1_6—C1_6	1.878 (14)
Cu14—S1_6	2.274 (4)	O1_6—C3_6	1.214 (11)
Cu14—S1A_5	2.29 (5)	O2_6—C3_6	1.222 (11)
Cu14—Cl13	2.303 (3)	N1_6—C2_6	1.558 (16)
Cu14—Cl11	2.682 (3)	N1_6—H1A_6	0.9100
Cu14—Cu15	3.032 (2)	N1_6—H1B_6	0.9100
Cu15—S1_5	2.15 (3)	N1_6—H1C_6	0.9100
Cu15—S1_4	2.233 (3)	C1_6—C2_6	1.502 (17)
Cu15—Cl14	2.310 (3)	C1_6—H1D_6	0.9900
Cu15—S1A_5	2.37 (4)	C1_6—H1E_6	0.9900
Cu16—Cl13	2.284 (3)	C2_6—C3_6	1.594 (16)
Cu16—Cl12	2.289 (3)	C2_6—H2_6	1.0000
Cu16—Cl14	2.341 (3)	O4_6—H4A_6	0.8401 (15)
Cu16—Cl17	2.604 (3)	O4_6—H4B_6	0.8400 (17)
Cl17—Cu20	2.293 (2)	S1A_6—C1A_6	1.877 (19)
Cl17—Cu17	2.358 (3)	O1A_6—C3A_6	1.211 (14)
Cl18—Cu18	2.292 (3)	O2A_6—C3A_6	1.214 (14)
Cl18—Cu17	2.312 (3)	N1A_6—C2A_6	1.57 (2)
Cl19—Cu19	2.284 (3)	N1A_6—H1AA_6	0.9100
Cl19—Cu17	2.300 (3)	N1A_6—H1AB_6	0.9100
Cl20—Cu28	2.277 (3)	N1A_6—H1AC_6	0.9100
Cl20—Cu27	2.298 (3)	C1A_6—C2A_6	1.59 (7)
Cl21—Cu21	2.524 (3)	C1A_6—H1BA_6	0.9900
Cl21—Cu18	2.767 (3)	C1A_6—H1BB_6	0.9900
Cl22—Cu28	2.295 (3)	C2A_6—C3A_6	1.61 (2)
Cl22—Cu21	2.363 (3)	C2A_6—H2A_6	1.0000
Cl22—Cu32^iv^	2.505 (3)	S1_7—C1_7	1.840 (9)
Cl23—Cu29	2.286 (3)	O1_7—C3_7	1.222 (10)
Cl23—Cu28	2.347 (3)	O2_7—C3_7	1.312 (11)
Cl24—Cu26	2.279 (3)	N1_7—C2_7	1.499 (11)
Cl24—Cu22	2.286 (2)	N1_7—H1A_7	0.9100
Cl25—Cu30	2.312 (2)	N1_7—H1B_7	0.9100
Cl25—Cu26	2.356 (3)	N1_7—H1C_7	0.9100
Cl25—Cu26^v^	2.570 (3)	C1_7—C2_7	1.493 (11)
Cl26—Cu29	2.740 (3)	C1_7—H1AA_7	0.9900
Cl27—Cu26	2.287 (3)	C1_7—H1AB_7	0.9900
Cl27—Cu23	2.378 (3)	C2_7—C3_7	1.500 (11)
Cl28—Cu25	2.463 (3)	C2_7—H2_7	1.0000
Cl29—Cu23	2.524 (3)	S1_8—C1_8	1.823 (11)
Cl30—Cu32	2.297 (3)	O1_8—C3_8	1.234 (13)
Cl30—Cu25	2.384 (3)	O2_8—C3_8	1.309 (13)
Cl31—Cu31	2.289 (3)	N1_8—C2_8	1.509 (13)
Cl31—Cu32	2.371 (3)	N1_8—H1A_8	0.9100
Cl31—Cu28^vi^	2.626 (3)	N1_8—H1B_8	0.9100
Cl32—Cu24	2.283 (3)	N1_8—H1C_8	0.9100
Cl32—Cu32	2.311 (3)	C1_8—C2_8	1.485 (14)
Cu17—Cl14	2.594 (3)	C1_8—H1AA_8	0.9900
Cu17—Cu20	2.9094 (19)	C1_8—H1AB_8	0.9900
Cu17—Cu19	3.0024 (18)	C2_8—C3_8	1.537 (14)
Cu17—Cu18	3.0272 (18)	C2_8—H2_8	1.0000
Cu18—S1_11	2.229 (3)	S1_9—C1_9	1.832 (8)
Cu18—S1_9	2.287 (3)	O1_9—C3_9	1.226 (11)
Cu18—Cu20	2.7830 (19)	O2_9—C3_9	1.252 (11)
Cu18—Cu21	2.9437 (19)	N1_9—C2_9	1.523 (12)
Cu18—Cu19	2.9469 (18)	N1_9—H1A_9	0.9100
Cu19—S1_12	2.259 (3)	N1_9—H1B_9	0.9100
Cu19—S1_11	2.284 (3)	N1_9—H1C_9	0.9100
Cu19—Cu20	2.7589 (18)	C1_9—C2_9	1.496 (11)
Cu19—Cu23	2.9070 (18)	C1_9—H1D_9	0.9900
Cu20—S1_9	2.217 (2)	C1_9—H1DE_9	0.9900
Cu20—S1_12	2.246 (2)	C2_9—C3_9	1.513 (11)
Cu20—Cu25	2.9624 (19)	C2_9—H2_9	1.0000
Cu21—S1_7	2.277 (3)	S1_10—C1_10	1.825 (8)
Cu21—S1_9	2.289 (3)	O1_10—C3_10	1.221 (11)
Cu21—Cu22	2.7944 (17)	O2_10—C3_10	1.270 (11)
Cu22—S1_11	2.221 (3)	N1_10—C2_10	1.487 (11)
Cu22—S1_7	2.268 (3)	N1_10—H1A_10	0.9100
Cu22—Cu30	2.6986 (18)	N1_10—H1B_10	0.9100
Cu22—Cu26	2.8853 (16)	N1_10—H1C_10	0.9100
Cu22—Cu23	2.9494 (17)	C1_10—C2_10	1.540 (11)
Cu23—S1_11	2.282 (3)	C1_10—H1D_10	0.9900
Cu23—S1_10	2.302 (3)	C1_10—H1DE_10	0.9900
Cu23—Cu31	2.882 (2)	C2_10—C3_10	1.538 (12)
Cu23—Cu30	3.030 (2)	C2_10—H2_10	1.0000
Cu24—S1_8	2.222 (3)	S1_11—C1_11	1.830 (10)
Cu24—S1_10	2.226 (3)	O1_11—C3_11	1.211 (13)
Cu24—Cu31	2.830 (2)	O2_11—C3_11	1.290 (12)
Cu24—Cu32	3.008 (2)	N1_11—C2_11	1.491 (11)
Cu25—S1_8	2.291 (3)	N1_11—H1A_11	0.9100
Cu25—S1_12	2.291 (3)	N1_11—H1B_11	0.9100
Cu25—Cu27	2.8418 (17)	N1_11—H1C_11	0.9100
Cu25—Cu31	2.959 (2)	C1_11—C2_11	1.501 (13)
Cu26—Cu30	2.8768 (19)	C1_11—H1AA_11	0.9900
Cu26—Cu26^v^	3.049 (2)	C1_11—H1AB_11	0.9900
Cu27—S1_9	2.224 (3)	C2_11—C3_11	1.489 (13)
Cu27—S1_8	2.230 (3)	C2_11—H2_11	1.0000
Cu27—Cu28	2.8866 (18)	S1_12—C1_12	1.823 (8)
Cu27—Cu29	2.901 (2)	O1_12—C3_12	1.226 (12)
Cu28—Cu32^iv^	3.0548 (18)	O2_12—C3_12	1.269 (12)
Cu29—S1_7	2.245 (3)	N1_12—C2_12	1.501 (11)
Cu29—S1_8	2.255 (3)	N1_12—H1A_12	0.9100
Cu30—S1_10	2.225 (2)	N1_12—H1B_12	0.9100
Cu30—S1_7	2.238 (2)	N1_12—H1C_12	0.9100
Cu31—S1_12	2.212 (3)	C1_12—C2_12	1.514 (12)
Cu31—S1_10	2.215 (3)	C1_12—H1AA_12	0.9900
Cu31—Cu32	3.036 (2)	C1_12—H1AB_12	0.9900
S1_1—C1_1	1.837 (19)	C2_12—C3_12	1.522 (12)
O1_1—C3_1	1.246 (16)	C2_12—H2_12	1.0000
O2_1—C3_1	1.272 (17)	O1_13—H1A_13	0.82 (3)
N1_1—C2_1	1.555 (17)	O1_13—H1B_13	0.83 (3)
N1_1—H1A_1	0.9100		
			
Cl3—Cu1—Cl1	117.68 (14)	Cl27—Cu26—Cu26^v^	131.07 (7)
Cl3—Cu1—Cl2	122.94 (12)	Cl25—Cu26—Cu26^v^	55.00 (7)
Cl1—Cu1—Cl2	112.72 (13)	Cl25^v^—Cu26—Cu26^v^	48.68 (7)
Cl3—Cu1—Cl6^i^	99.92 (10)	Cu30—Cu26—Cu26^v^	104.12 (7)
Cl1—Cu1—Cl6^i^	103.06 (11)	Cu22—Cu26—Cu26^v^	133.25 (5)
Cl2—Cu1—Cl6^i^	93.04 (10)	S1_9—Cu27—S1_8	133.93 (11)
Cl3—Cu1—Cu4	99.55 (9)	S1_9—Cu27—Cl20	108.72 (10)
Cl1—Cu1—Cu4	94.11 (10)	S1_8—Cu27—Cl20	116.73 (11)
Cl2—Cu1—Cu4	51.07 (7)	S1_9—Cu27—Cu25	102.03 (8)
Cl6^i^—Cu1—Cu4	144.07 (9)	S1_8—Cu27—Cu25	52.02 (7)
Cl3—Cu1—Cu2	95.34 (9)	Cl20—Cu27—Cu25	131.21 (9)
Cl1—Cu1—Cu2	48.02 (8)	S1_9—Cu27—Cu28	98.04 (8)
Cl2—Cu1—Cu2	99.09 (8)	S1_8—Cu27—Cu28	104.65 (8)
Cl6^i^—Cu1—Cu2	151.08 (8)	Cl20—Cu27—Cu28	50.55 (7)
Cu4—Cu1—Cu2	54.66 (5)	Cu25—Cu27—Cu28	156.37 (6)
S1_2—Cu2—S1_3	123.71 (12)	S1_9—Cu27—Cu29	110.13 (8)
S1_2—Cu2—Cl1	122.58 (15)	S1_8—Cu27—Cu29	50.06 (7)
S1_3—Cu2—Cl1	112.23 (14)	Cl20—Cu27—Cu29	107.03 (8)
S1_2—Cu2—Cu4	104.48 (9)	Cu25—Cu27—Cu29	96.36 (5)
S1_3—Cu2—Cu4	51.76 (8)	Cu28—Cu27—Cu29	64.77 (5)
Cl1—Cu2—Cu4	99.56 (10)	Cl20—Cu28—Cl22	128.41 (11)
S1_2—Cu2—Cu3	48.59 (8)	Cl20—Cu28—Cl23	115.36 (11)
S1_3—Cu2—Cu3	105.17 (9)	Cl22—Cu28—Cl23	112.48 (10)
Cl1—Cu2—Cu3	107.37 (11)	Cl20—Cu28—Cl31^iv^	97.52 (9)
Cu4—Cu2—Cu3	61.16 (5)	Cl22—Cu28—Cl31^iv^	95.60 (10)
S1_2—Cu2—Cu6	94.88 (9)	Cl23—Cu28—Cl31^iv^	96.03 (9)
S1_3—Cu2—Cu6	49.06 (8)	Cl20—Cu28—Cu27	51.21 (7)
Cl1—Cu2—Cu6	134.15 (11)	Cl22—Cu28—Cu27	105.10 (8)
Cu4—Cu2—Cu6	94.76 (6)	Cl23—Cu28—Cu27	97.34 (7)
Cu3—Cu2—Cu6	117.65 (6)	Cl31^iv^—Cu28—Cu27	148.71 (8)
S1_2—Cu2—Cu1	103.43 (9)	Cl20—Cu28—Cu32^iv^	103.79 (8)
S1_3—Cu2—Cu1	102.55 (9)	Cl22—Cu28—Cu32^iv^	53.59 (7)
Cl1—Cu2—Cu1	49.07 (8)	Cl23—Cu28—Cu32^iv^	130.85 (8)
Cu4—Cu2—Cu1	59.92 (5)	Cl31^iv^—Cu28—Cu32^iv^	48.61 (7)
Cu3—Cu2—Cu1	63.67 (5)	Cu27—Cu28—Cu32^iv^	130.99 (6)
Cu6—Cu2—Cu1	151.60 (7)	S1_7—Cu29—S1_8	125.43 (11)
S1_2—Cu3—S1_5	128.6 (7)	S1_7—Cu29—Cl23	117.35 (11)
S1_2—Cu3—S1A_5	130.5 (10)	S1_8—Cu29—Cl23	111.70 (11)
S1_2—Cu3—Cl3	112.21 (13)	S1_7—Cu29—Cl26	98.46 (10)
S1_5—Cu3—Cl3	112.2 (7)	S1_8—Cu29—Cl26	96.90 (10)
S1A_5—Cu3—Cl3	107.6 (10)	Cl23—Cu29—Cl26	98.08 (10)
S1_2—Cu3—Cl15	102.54 (12)	S1_7—Cu29—Cu27	99.99 (9)
S1_5—Cu3—Cl15	98.3 (9)	S1_8—Cu29—Cu27	49.32 (7)
S1A_5—Cu3—Cl15	102.4 (11)	Cl23—Cu29—Cu27	98.35 (9)
Cl3—Cu3—Cl15	94.60 (11)	Cl26—Cu29—Cu27	146.06 (8)
S1_2—Cu3—Cu12	50.35 (8)	S1_10—Cu30—S1_7	130.46 (10)
S1_5—Cu3—Cu12	103.3 (8)	S1_10—Cu30—Cl25	117.32 (10)
S1A_5—Cu3—Cu12	108.7 (10)	S1_7—Cu30—Cl25	112.21 (9)
Cl3—Cu3—Cu12	140.59 (10)	S1_10—Cu30—Cu22	106.53 (8)
Cl15—Cu3—Cu12	62.95 (8)	S1_7—Cu30—Cu22	53.71 (7)
S1_2—Cu3—Cu15	108.40 (9)	Cl25—Cu30—Cu22	108.82 (8)
S1_5—Cu3—Cu15	46.9 (8)	S1_10—Cu30—Cu26	108.00 (9)
S1A_5—Cu3—Cu15	52.6 (11)	S1_7—Cu30—Cu26	100.28 (8)
Cl3—Cu3—Cu15	136.06 (11)	Cl25—Cu30—Cu26	52.66 (7)
Cl15—Cu3—Cu15	60.23 (9)	Cu22—Cu30—Cu26	62.23 (5)
Cu12—Cu3—Cu15	62.43 (5)	S1_10—Cu30—Cu23	49.07 (8)
S1_2—Cu3—Cu4	98.75 (9)	S1_7—Cu30—Cu23	109.07 (8)
S1_5—Cu3—Cu4	50.8 (9)	Cl25—Cu30—Cu23	113.25 (8)
S1A_5—Cu3—Cu4	46.8 (11)	Cu22—Cu30—Cu23	61.65 (4)
Cl3—Cu3—Cu4	97.81 (9)	Cu26—Cu30—Cu23	70.35 (5)
Cl15—Cu3—Cu4	149.09 (10)	S1_12—Cu31—S1_10	132.71 (10)
Cu12—Cu3—Cu4	118.22 (6)	S1_12—Cu31—Cl31	115.40 (10)
Cu15—Cu3—Cu4	91.90 (6)	S1_10—Cu31—Cl31	111.83 (10)
S1_2—Cu3—Cu2	48.31 (8)	S1_12—Cu31—Cu24	111.17 (9)
S1_5—Cu3—Cu2	102.7 (8)	S1_10—Cu31—Cu24	50.59 (8)
S1A_5—Cu3—Cu2	100.2 (11)	Cl31—Cu31—Cu24	106.54 (9)
Cl3—Cu3—Cu2	95.96 (10)	S1_12—Cu31—Cu23	98.79 (9)
Cl15—Cu3—Cu2	150.81 (10)	S1_10—Cu31—Cu23	51.70 (8)
Cu12—Cu3—Cu2	92.38 (5)	Cl31—Cu31—Cu23	127.11 (9)
Cu15—Cu3—Cu2	123.98 (7)	Cu24—Cu31—Cu23	96.04 (6)
Cu4—Cu3—Cu2	55.23 (5)	S1_12—Cu31—Cu25	50.10 (8)
S1A_5—Cu4—S1_3	133.8 (9)	S1_10—Cu31—Cu25	108.12 (9)
S1_3—Cu4—S1_5	129.4 (7)	Cl31—Cu31—Cu25	112.92 (9)
S1A_5—Cu4—Cl2	107.0 (9)	Cu24—Cu31—Cu25	64.41 (5)
S1_3—Cu4—Cl2	119.20 (10)	Cu23—Cu31—Cu25	119.97 (5)
S1_5—Cu4—Cl2	111.4 (7)	S1_12—Cu31—Cu32	110.60 (9)
S1A_5—Cu4—Cu2	112.0 (12)	S1_10—Cu31—Cu32	97.10 (9)
S1_3—Cu4—Cu2	53.22 (9)	Cl31—Cu31—Cu32	50.52 (8)
S1_5—Cu4—Cu2	109.7 (8)	Cu24—Cu31—Cu32	61.59 (5)
Cl2—Cu4—Cu2	109.62 (9)	Cu23—Cu31—Cu32	147.74 (6)
S1A_5—Cu4—Cu1	99.0 (14)	Cu25—Cu31—Cu32	73.34 (5)
S1_3—Cu4—Cu1	108.44 (10)	Cl30—Cu32—Cl32	116.45 (11)
S1_5—Cu4—Cu1	101.5 (10)	Cl30—Cu32—Cl31	110.64 (11)
Cl2—Cu4—Cu1	52.21 (8)	Cl32—Cu32—Cl31	120.05 (11)
Cu2—Cu4—Cu1	65.42 (5)	Cl30—Cu32—Cl22^vi^	103.22 (10)
S1A_5—Cu4—Cu3	50.7 (14)	Cl32—Cu32—Cl22^vi^	105.85 (9)
S1_3—Cu4—Cu3	108.56 (9)	Cl31—Cu32—Cl22^vi^	96.96 (9)
S1_5—Cu4—Cu3	49.8 (10)	Cl30—Cu32—Cu24	89.52 (8)
Cl2—Cu4—Cu3	109.93 (9)	Cl32—Cu32—Cu24	48.68 (7)
Cu2—Cu4—Cu3	63.62 (5)	Cl31—Cu32—Cu24	99.14 (8)
Cu1—Cu4—Cu3	66.32 (5)	Cl22^vi^—Cu32—Cu24	154.43 (8)
S1A_5—Cu4—Cu7	102.0 (13)	Cl30—Cu32—Cu31	88.56 (8)
S1_3—Cu4—Cu7	48.16 (9)	Cl32—Cu32—Cu31	97.77 (8)
S1_5—Cu4—Cu7	98.8 (9)	Cl31—Cu32—Cu31	48.18 (7)
Cl2—Cu4—Cu7	129.50 (9)	Cl22^vi^—Cu32—Cu31	144.87 (9)
Cu2—Cu4—Cu7	96.09 (6)	Cu24—Cu32—Cu31	55.83 (5)
Cu1—Cu4—Cu7	156.21 (6)	Cl30—Cu32—Cu28^vi^	136.35 (8)
Cu3—Cu4—Cu7	120.44 (6)	Cl32—Cu32—Cu28^vi^	104.04 (7)
S1_1—Cu5—S1_2	137.8 (6)	Cl31—Cu32—Cu28^vi^	56.20 (7)
S1_1—Cu5—Cl4	108.5 (6)	Cl22^vi^—Cu32—Cu28^vi^	47.49 (7)
S1_2—Cu5—Cl4	113.32 (13)	Cu24—Cu32—Cu28^vi^	131.43 (6)
S1_2—Cu5—S1A_1	133.8 (12)	Cu31—Cu32—Cu28^vi^	102.00 (6)
Cl4—Cu5—S1A_1	112.7 (11)	Cu2—Cl1—Cu1	82.91 (11)
S1_1—Cu5—Cu6	53.9 (6)	Cu4—Cl2—Cu1	76.72 (9)
S1_2—Cu5—Cu6	103.08 (9)	Cu4—Cl2—Cu11^i^	149.49 (12)
Cl4—Cu5—Cu6	124.94 (9)	Cu1—Cl2—Cu11^i^	82.50 (9)
S1A_1—Cu5—Cu6	51.0 (13)	Cu1—Cl3—Cu3	88.88 (11)
S1_1—Cu5—Cu12	110.2 (8)	Cu11—Cl4—Cu5	78.80 (10)
S1_2—Cu5—Cu12	52.42 (8)	Cu10—Cl6—Cu11	80.86 (10)
Cl4—Cu5—Cu12	107.01 (9)	Cu10—Cl6—Cu1^iii^	149.02 (12)
S1A_1—Cu5—Cu12	109.1 (15)	Cu11—Cl6—Cu1^iii^	83.18 (10)
Cu6—Cu5—Cu12	127.98 (6)	Cu9—Cl7—Cu6	93.22 (12)
S1_1—Cu5—Cu10	50.7 (7)	Cu9—Cl7—Cu9^ii^	78.14 (12)
S1_2—Cu5—Cu10	110.17 (9)	Cu6—Cl7—Cu9^ii^	149.00 (14)
Cl4—Cu5—Cu10	106.60 (9)	Cu12—Cl8—Cu11	82.02 (11)
S1A_1—Cu5—Cu10	51.3 (15)	Cu8—Cl9—Cu9	82.54 (13)
Cu6—Cu5—Cu10	97.24 (7)	Cu9—Cl10—Cu7	87.41 (11)
Cu12—Cu5—Cu10	62.47 (5)	Cu16—Cl12—Cu13	83.23 (10)
S1_1—Cu5—Cu11	98.8 (6)	Cu16—Cl13—Cu14	86.53 (12)
S1_2—Cu5—Cu11	103.63 (9)	Cu15—Cl14—Cu16	82.98 (10)
Cl4—Cu5—Cu11	50.23 (8)	Cu15—Cl14—Cu17	166.76 (15)
S1A_1—Cu5—Cu11	102.2 (12)	Cu16—Cl14—Cu17	85.33 (10)
Cu6—Cu5—Cu11	151.51 (7)	C1_1—S1_1—Cu5	105.2 (13)
Cu12—Cu5—Cu11	64.50 (5)	C1_1—S1_1—Cu10	111.8 (16)
Cu10—Cu5—Cu11	64.12 (5)	Cu5—S1_1—Cu10	79.1 (8)
S1A_1—Cu6—S1_3	122.9 (14)	C1_1—S1_1—Cu8	118.9 (14)
S1_1—Cu6—S1_3	125.8 (6)	Cu5—S1_1—Cu8	135.7 (10)
S1A_1—Cu6—Cl7	104.9 (13)	Cu10—S1_1—Cu8	86.7 (11)
S1_1—Cu6—Cl7	105.6 (6)	C1_1—S1_1—Cu6	109.9 (18)
S1_3—Cu6—Cl7	110.36 (11)	Cu5—S1_1—Cu6	75.0 (9)
S1A_1—Cu6—Cl5	118.6 (16)	Cu10—S1_1—Cu6	135.3 (10)
S1_1—Cu6—Cl5	114.3 (8)	Cu8—S1_1—Cu6	87.1 (7)
S1_3—Cu6—Cl5	100.06 (13)	C2_1—N1_1—H1A_1	109.5
Cl7—Cu6—Cl5	97.02 (13)	C2_1—N1_1—H1B_1	109.5
S1A_1—Cu6—Cu5	54.2 (15)	H1A_1—N1_1—H1B_1	109.5
S1_1—Cu6—Cu5	51.2 (7)	C2_1—N1_1—H1C_1	109.5
S1_3—Cu6—Cu5	106.91 (8)	H1A_1—N1_1—H1C_1	109.5
Cl7—Cu6—Cu5	142.66 (9)	H1B_1—N1_1—H1C_1	109.5
Cl5—Cu6—Cu5	73.96 (9)	C2_1—C1_1—S1_1	110.9 (16)
S1A_1—Cu6—Cu2	114.2 (13)	C2_1—C1_1—H1D_1	109.5
S1_1—Cu6—Cu2	113.3 (6)	S1_1—C1_1—H1D_1	109.5
S1_3—Cu6—Cu2	48.17 (9)	C2_1—C1_1—H1E_1	109.5
Cl7—Cu6—Cu2	140.86 (10)	S1_1—C1_1—H1E_1	109.5
Cl5—Cu6—Cu2	63.36 (10)	H1D_1—C1_1—H1E_1	108.0
Cu5—Cu6—Cu2	67.72 (5)	C1_1—C2_1—C3_1	116.8 (16)
S1_3—Cu7—S1A_6	130.5 (8)	C1_1—C2_1—N1_1	108.0 (19)
S1_3—Cu7—S1_6	131.18 (19)	C3_1—C2_1—N1_1	106.3 (13)
S1_3—Cu7—Cl10	107.14 (12)	C1_1—C2_1—H2_1	108.5
S1A_6—Cu7—Cl10	116.4 (7)	C3_1—C2_1—H2_1	108.5
S1_6—Cu7—Cl10	115.66 (18)	N1_1—C2_1—H2_1	108.5
S1_3—Cu7—Cu14	107.82 (9)	O1_1—C3_1—O2_1	132.1 (17)
S1A_6—Cu7—Cu14	50.8 (4)	O1_1—C3_1—C2_1	119.3 (15)
S1_6—Cu7—Cu14	51.27 (12)	O2_1—C3_1—C2_1	108.3 (15)
Cl10—Cu7—Cu14	137.58 (12)	C1A_1—S1A_1—Cu8	114 (3)
S1_3—Cu7—Cu8	102.57 (10)	C1A_1—S1A_1—Cu6	112 (4)
S1A_6—Cu7—Cu8	49.6 (4)	Cu8—S1A_1—Cu6	93.7 (18)
S1_6—Cu7—Cu8	49.59 (12)	C1A_1—S1A_1—Cu10	105 (3)
Cl10—Cu7—Cu8	99.43 (10)	Cu8—S1A_1—Cu10	90 (2)
Cu14—Cu7—Cu8	95.69 (7)	Cu6—S1A_1—Cu10	138 (2)
S1_3—Cu7—Cu4	47.95 (7)	C1A_1—S1A_1—Cu5	105 (3)
S1A_6—Cu7—Cu4	107.4 (6)	Cu8—S1A_1—Cu5	141 (2)
S1_6—Cu7—Cu4	108.10 (14)	Cu6—S1A_1—Cu5	74.9 (19)
Cl10—Cu7—Cu4	132.80 (10)	Cu10—S1A_1—Cu5	76.4 (16)
Cu14—Cu7—Cu4	63.46 (5)	C2A_1—N1A_1—H1AA_1	109.5
Cu8—Cu7—Cu4	122.77 (7)	C2A_1—N1A_1—H1AB_1	109.5
S1A_1—Cu8—S1A_6	125.1 (15)	H1AA_1—N1A_1—H1AB_1	109.5
S1A_1—Cu8—Cl9	122.4 (13)	C2A_1—N1A_1—H1AC_1	109.5
S1A_6—Cu8—Cl9	111.3 (8)	H1AA_1—N1A_1—H1AC_1	109.5
S1_6—Cu8—Cl9	110.5 (2)	H1AB_1—N1A_1—H1AC_1	109.5
S1_6—Cu8—S1_1	126.9 (6)	C2A_1—C1A_1—S1A_1	131 (6)
Cl9—Cu8—S1_1	121.9 (6)	C2A_1—C1A_1—H1BA_1	104.4
S1A_1—Cu8—Cu7	104.9 (13)	S1A_1—C1A_1—H1BA_1	104.4
S1A_6—Cu8—Cu7	49.2 (4)	C2A_1—C1A_1—H1BB_1	104.4
S1_6—Cu8—Cu7	49.50 (12)	S1A_1—C1A_1—H1BB_1	104.4
Cl9—Cu8—Cu7	102.89 (12)	H1BA_1—C1A_1—H1BB_1	105.6
S1_1—Cu8—Cu7	107.2 (6)	C1A_1—C2A_1—N1A_1	103 (4)
S1A_1—Cu8—Cu9	101.2 (16)	C1A_1—C2A_1—C3A_1	130 (5)
S1A_6—Cu8—Cu9	104.4 (5)	N1A_1—C2A_1—C3A_1	113 (3)
S1_6—Cu8—Cu9	104.20 (14)	C1A_1—C2A_1—H2A_1	102.5
Cl9—Cu8—Cu9	50.34 (10)	N1A_1—C2A_1—H2A_1	102.5
S1_1—Cu8—Cu9	102.6 (7)	C3A_1—C2A_1—H2A_1	102.5
Cu7—Cu8—Cu9	64.62 (6)	O1A_1—C3A_1—O2A_1	118 (5)
Cl7—Cu9—Cl10	124.68 (14)	O1A_1—C3A_1—C2A_1	125 (6)
Cl7—Cu9—Cl9	116.46 (14)	O2A_1—C3A_1—C2A_1	117 (4)
Cl10—Cu9—Cl9	110.10 (14)	C1_2—S1_2—Cu5	114.4 (5)
Cl7—Cu9—Cl7^ii^	93.99 (13)	C1_2—S1_2—Cu2	113.3 (4)
Cl10—Cu9—Cl7^ii^	104.96 (11)	Cu5—S1_2—Cu2	91.98 (12)
Cl9—Cu9—Cl7^ii^	101.10 (12)	C1_2—S1_2—Cu3	108.5 (5)
Cl7—Cu9—Cu8	94.09 (9)	Cu5—S1_2—Cu3	134.92 (14)
Cl10—Cu9—Cu8	96.49 (9)	Cu2—S1_2—Cu3	83.10 (11)
Cl9—Cu9—Cu8	47.12 (9)	C1_2—S1_2—Cu12	106.0 (4)
Cl7^ii^—Cu9—Cu8	146.94 (10)	Cu5—S1_2—Cu12	76.15 (10)
S1_1—Cu10—S1_4	136.2 (5)	Cu2—S1_2—Cu12	140.31 (15)
S1_4—Cu10—S1A_1	133.0 (10)	Cu3—S1_2—Cu12	79.60 (10)
S1_1—Cu10—Cl6	112.6 (5)	C2_2—N1_2—H1A_2	109.5
S1_4—Cu10—Cl6	107.80 (11)	C2_2—N1_2—H1B_2	109.5
S1A_1—Cu10—Cl6	116.9 (11)	H1A_2—N1_2—H1B_2	109.5
S1_1—Cu10—Cu5	50.2 (8)	C2_2—N1_2—H1C_2	109.5
S1_4—Cu10—Cu5	104.17 (10)	H1A_2—N1_2—H1C_2	109.5
S1A_1—Cu10—Cu5	52.3 (16)	H1B_2—N1_2—H1C_2	109.5
Cl6—Cu10—Cu5	104.43 (9)	C2_2—C1_2—S1_2	110.6 (9)
S1_1—Cu10—Cu12	105.3 (7)	C2_2—C1_2—H1AA_2	109.5
S1_4—Cu10—Cu12	49.87 (9)	S1_2—C1_2—H1AA_2	109.5
S1A_1—Cu10—Cu12	105.7 (14)	C2_2—C1_2—H1AB_2	109.5
Cl6—Cu10—Cu12	100.85 (10)	S1_2—C1_2—H1AB_2	109.5
Cu5—Cu10—Cu12	57.78 (5)	H1AA_2—C1_2—H1AB_2	108.1
S1_1—Cu10—Cu13	109.5 (6)	C3_2—C2_2—C1_2	108.3 (13)
S1_4—Cu10—Cu13	49.00 (9)	C3_2—C2_2—N1_2	109.0 (12)
S1A_1—Cu10—Cu13	104.8 (14)	C1_2—C2_2—N1_2	116.2 (12)
Cl6—Cu10—Cu13	130.52 (9)	C3_2—C2_2—H2_2	107.7
Cu5—Cu10—Cu13	122.34 (6)	C1_2—C2_2—H2_2	107.7
Cu12—Cu10—Cu13	91.83 (6)	N1_2—C2_2—H2_2	107.7
S1_1—Cu10—Cu11	94.3 (7)	O1_2—C3_2—O2_2	124.6 (16)
S1_4—Cu10—Cu11	99.32 (10)	O1_2—C3_2—C2_2	116.7 (15)
S1A_1—Cu10—Cu11	98.7 (15)	O2_2—C3_2—C2_2	117.3 (14)
Cl6—Cu10—Cu11	49.64 (9)	C1_3—S1_3—Cu4	117.3 (3)
Cu5—Cu10—Cu11	58.87 (5)	C1_3—S1_3—Cu7	109.7 (4)
Cu12—Cu10—Cu11	61.10 (5)	Cu4—S1_3—Cu7	83.88 (11)
Cu13—Cu10—Cu11	148.31 (7)	C1_3—S1_3—Cu2	108.2 (4)
Cl4—Cu11—Cl8	120.14 (14)	Cu4—S1_3—Cu2	75.02 (10)
Cl4—Cu11—Cl6	124.44 (13)	Cu7—S1_3—Cu2	141.82 (12)
Cl8—Cu11—Cl6	110.98 (12)	C1_3—S1_3—Cu6	101.9 (3)
Cl4—Cu11—Cl2^iii^	97.20 (10)	Cu4—S1_3—Cu6	139.18 (12)
Cl8—Cu11—Cl2^iii^	101.21 (10)	Cu7—S1_3—Cu6	93.65 (12)
Cl6—Cu11—Cl2^iii^	92.76 (10)	Cu2—S1_3—Cu6	82.77 (11)
Cl4—Cu11—Cu5	50.96 (8)	C2_3—N1_3—H1A_3	109.5
Cl8—Cu11—Cu5	99.32 (9)	C2_3—N1_3—H1B_3	109.5
Cl6—Cu11—Cu5	102.54 (9)	H1A_3—N1_3—H1B_3	109.5
Cl2^iii^—Cu11—Cu5	147.94 (8)	C2_3—N1_3—H1C_3	109.5
Cl4—Cu11—Cu12	99.98 (8)	H1A_3—N1_3—H1C_3	109.5
Cl8—Cu11—Cu12	48.83 (8)	H1B_3—N1_3—H1C_3	109.5
Cl6—Cu11—Cu12	97.48 (8)	C2_3—C1_3—S1_3	116.5 (6)
Cl2^iii^—Cu11—Cu12	150.02 (8)	C2_3—C1_3—H1D_3	108.2
Cu5—Cu11—Cu12	55.88 (4)	S1_3—C1_3—H1D_3	108.2
Cl4—Cu11—Cu10	100.91 (10)	C2_3—C1_3—H1DE_3	108.2
Cl8—Cu11—Cu10	98.19 (10)	S1_3—C1_3—H1DE_3	108.2
Cl6—Cu11—Cu10	49.50 (7)	H1D_3—C1_3—H1DE_3	107.3
Cl2^iii^—Cu11—Cu10	141.90 (9)	N1_3—C2_3—C3_3	109.3 (8)
Cu5—Cu11—Cu10	57.01 (5)	N1_3—C2_3—C1_3	111.8 (9)
Cu12—Cu11—Cu10	57.32 (5)	C3_3—C2_3—C1_3	109.5 (7)
S1_4—Cu12—S1_2	133.70 (12)	N1_3—C2_3—H2_3	108.7
S1_4—Cu12—Cl8	110.34 (12)	C3_3—C2_3—H2_3	108.7
S1_2—Cu12—Cl8	113.81 (13)	C1_3—C2_3—H2_3	108.7
S1_4—Cu12—Cu5	105.62 (9)	O2_3—C3_3—O1_3	126.2 (9)
S1_2—Cu12—Cu5	51.44 (8)	O2_3—C3_3—C2_3	118.0 (9)
Cl8—Cu12—Cu5	103.03 (10)	O1_3—C3_3—C2_3	115.8 (9)
S1_4—Cu12—Cu3	103.80 (9)	C1_4—S1_4—Cu15	118.7 (3)
S1_2—Cu12—Cu3	50.04 (8)	C1_4—S1_4—Cu10	103.6 (3)
Cl8—Cu12—Cu3	135.56 (11)	Cu15—S1_4—Cu10	137.67 (13)
Cu5—Cu12—Cu3	94.01 (5)	C1_4—S1_4—Cu12	112.7 (5)
S1_4—Cu12—Cu10	49.43 (8)	Cu15—S1_4—Cu12	84.27 (12)
S1_2—Cu12—Cu10	106.76 (9)	Cu10—S1_4—Cu12	80.70 (10)
Cl8—Cu12—Cu10	102.22 (10)	C1_4—S1_4—Cu13	107.8 (5)
Cu5—Cu12—Cu10	59.75 (5)	Cu15—S1_4—Cu13	82.38 (12)
Cu3—Cu12—Cu10	121.68 (7)	Cu10—S1_4—Cu13	83.61 (12)
S1_4—Cu12—Cu15	47.61 (8)	Cu12—S1_4—Cu13	139.02 (13)
S1_2—Cu12—Cu15	105.08 (9)	C2_4—N1_4—H1A_4	109.5
Cl8—Cu12—Cu15	133.43 (10)	C2_4—N1_4—H1B_4	109.5
Cu5—Cu12—Cu15	121.35 (6)	H1A_4—N1_4—H1B_4	109.5
Cu3—Cu12—Cu15	59.18 (5)	C2_4—N1_4—H1C_4	109.5
Cu10—Cu12—Cu15	89.61 (6)	H1A_4—N1_4—H1C_4	109.5
S1_4—Cu12—Cu11	99.38 (8)	H1B_4—N1_4—H1C_4	109.5
S1_2—Cu12—Cu11	98.90 (8)	C2_4—C1_4—S1_4	113.4 (7)
Cl8—Cu12—Cu11	49.15 (8)	C2_4—C1_4—H1AA_4	108.9
Cu5—Cu12—Cu11	59.63 (4)	S1_4—C1_4—H1AA_4	108.9
Cu3—Cu12—Cu11	148.94 (6)	C2_4—C1_4—H1AB_4	108.9
Cu10—Cu12—Cu11	61.58 (5)	S1_4—C1_4—H1AB_4	108.9
Cu15—Cu12—Cu11	146.99 (7)	H1AA_4—C1_4—H1AB_4	107.7
S1A_6—Cu13—S1_4	124.9 (8)	C3_4—C2_4—N1_4	107.4 (9)
S1_6—Cu13—S1_4	125.68 (18)	C3_4—C2_4—C1_4	116.4 (9)
S1A_6—Cu13—Cl12	125.2 (7)	N1_4—C2_4—C1_4	109.8 (10)
S1_6—Cu13—Cl12	124.64 (19)	C3_4—C2_4—H2_4	107.6
S1_4—Cu13—Cl12	107.25 (12)	N1_4—C2_4—H2_4	107.6
S1A_6—Cu13—C1A_6	45.8 (7)	C1_4—C2_4—H2_4	107.6
S1_4—Cu13—C1A_6	167.9 (12)	O1_4—C3_4—O2_4	125.2 (11)
Cl12—Cu13—C1A_6	80.0 (9)	O1_4—C3_4—C2_4	121.7 (12)
S1A_6—Cu13—Cu14	49.0 (4)	O2_4—C3_4—C2_4	113.0 (10)
S1_6—Cu13—Cu14	49.42 (12)	C1_5—S1_5—Cu15	111 (2)
S1_4—Cu13—Cu14	103.76 (9)	C1_5—S1_5—Cu14	117.0 (18)
Cl12—Cu13—Cu14	107.28 (10)	Cu15—S1_5—Cu14	87.9 (14)
C1A_6—Cu13—Cu14	64.4 (12)	C1_5—S1_5—Cu3	100.6 (18)
S1A_6—Cu13—Cu15	104.1 (6)	Cu15—S1_5—Cu3	82.7 (11)
S1_6—Cu13—Cu15	104.82 (15)	Cu14—S1_5—Cu3	142.1 (12)
S1_4—Cu13—Cu15	47.94 (8)	C1_5—S1_5—Cu4	106.3 (16)
Cl12—Cu13—Cu15	98.45 (9)	Cu15—S1_5—Cu4	141.0 (12)
C1A_6—Cu13—Cu15	122.3 (13)	Cu14—S1_5—Cu4	85.5 (12)
Cu14—Cu13—Cu15	61.30 (5)	Cu3—S1_5—Cu4	79.4 (12)
S1A_6—Cu13—Cu10	96.7 (6)	C2_5—N1_5—H1A_5	109.5
S1_6—Cu13—Cu10	97.03 (15)	C2_5—N1_5—H1B_5	109.5
S1_4—Cu13—Cu10	47.39 (8)	H1A_5—N1_5—H1B_5	109.5
Cl12—Cu13—Cu10	133.71 (11)	C2_5—N1_5—H1C_5	109.5
C1A_6—Cu13—Cu10	133.6 (10)	H1A_5—N1_5—H1C_5	109.5
Cu14—Cu13—Cu10	115.71 (6)	H1B_5—N1_5—H1C_5	109.5
Cu15—Cu13—Cu10	87.99 (6)	C2_5—C1_5—S1_5	119.5 (19)
S1A_6—Cu13—Cu16	103.3 (5)	C2_5—C1_5—H1AA_5	107.4
S1_6—Cu13—Cu16	103.28 (13)	S1_5—C1_5—H1AA_5	107.4
S1_4—Cu13—Cu16	99.36 (9)	C2_5—C1_5—H1AB_5	107.4
Cl12—Cu13—Cu16	48.22 (8)	S1_5—C1_5—H1AB_5	107.4
C1A_6—Cu13—Cu16	78.0 (11)	H1AA_5—C1_5—H1AB_5	107.0
Cu14—Cu13—Cu16	63.01 (5)	C1_5—C2_5—C3_5	111.9 (17)
Cu15—Cu13—Cu16	61.47 (5)	C1_5—C2_5—N1_5	111.8 (18)
Cu10—Cu13—Cu16	146.63 (8)	C3_5—C2_5—N1_5	111.4 (13)
S1_5—Cu14—S1_6	127.7 (7)	C1_5—C2_5—H2_5	107.1
S1A_6—Cu14—S1A_5	129.3 (12)	C3_5—C2_5—H2_5	107.2
S1_5—Cu14—Cl13	118.0 (8)	N1_5—C2_5—H2_5	107.2
S1A_6—Cu14—Cl13	108.2 (7)	O1_5—C3_5—O2_5	123.5 (17)
S1_6—Cu14—Cl13	107.55 (18)	O1_5—C3_5—C2_5	117.9 (14)
S1A_5—Cu14—Cl13	118.5 (10)	O2_5—C3_5—C2_5	118.2 (16)
S1_5—Cu14—Cl11	102.7 (8)	C1A_5—S1A_5—Cu4	114 (2)
S1A_6—Cu14—Cl11	99.9 (5)	C1A_5—S1A_5—Cu3	99 (2)
S1_6—Cu14—Cl11	99.65 (14)	Cu4—S1A_5—Cu3	82.5 (16)
S1A_5—Cu14—Cl11	97.1 (11)	C1A_5—S1A_5—Cu14	123 (2)
Cl13—Cu14—Cl11	92.35 (11)	Cu4—S1A_5—Cu14	87.6 (17)
S1_5—Cu14—Cu7	104.3 (9)	Cu3—S1A_5—Cu14	136.8 (15)
S1A_6—Cu14—Cu7	50.2 (4)	C1A_5—S1A_5—Cu15	107 (2)
S1_6—Cu14—Cu7	50.40 (12)	Cu4—S1A_5—Cu15	136.2 (15)
S1A_5—Cu14—Cu7	102.0 (11)	Cu3—S1A_5—Cu15	77.7 (13)
Cl13—Cu14—Cu7	134.18 (11)	Cu14—S1A_5—Cu15	81.2 (15)
Cl11—Cu14—Cu7	60.45 (8)	C2A_5—N1A_5—H1A1_5	109.5
S1_5—Cu14—Cu13	101.3 (7)	C2A_5—N1A_5—H1A2_5	109.5
S1A_6—Cu14—Cu13	48.2 (5)	H1A1_5—N1A_5—H1A2_5	109.5
S1_6—Cu14—Cu13	48.36 (12)	C2A_5—N1A_5—H1A3_5	109.5
S1A_5—Cu14—Cu13	106.6 (10)	H1A1_5—N1A_5—H1A3_5	109.5
Cl13—Cu14—Cu13	94.96 (10)	H1A2_5—N1A_5—H1A3_5	109.5
Cl11—Cu14—Cu13	147.85 (9)	C2A_5—C1A_5—S1A_5	99 (2)
Cu7—Cu14—Cu13	93.02 (6)	C2A_5—C1A_5—H1AC_5	112.0
S1_5—Cu14—Cu15	45.0 (8)	S1A_5—C1A_5—H1AC_5	112.0
S1A_6—Cu14—Cu15	101.9 (6)	C2A_5—C1A_5—H1AD_5	112.0
S1_6—Cu14—Cu15	102.33 (15)	S1A_5—C1A_5—H1AD_5	112.0
S1A_5—Cu14—Cu15	50.6 (11)	H1AC_5—C1A_5—H1AD_5	109.7
Cl13—Cu14—Cu15	103.12 (10)	N1A_5—C2A_5—C3A_5	116 (2)
Cl11—Cu14—Cu15	147.70 (9)	N1A_5—C2A_5—C1A_5	108 (2)
Cu7—Cu14—Cu15	119.59 (7)	C3A_5—C2A_5—C1A_5	99 (2)
Cu13—Cu14—Cu15	59.59 (5)	N1A_5—C2A_5—H2A_5	111.2
S1_5—Cu15—S1_4	129.1 (7)	C3A_5—C2A_5—H2A_5	111.2
S1_5—Cu15—Cl14	106.9 (8)	C1A_5—C2A_5—H2A_5	111.2
S1_4—Cu15—Cl14	120.52 (11)	O2A_5—C3A_5—O1A_5	128 (3)
S1_4—Cu15—S1A_5	129.8 (9)	O2A_5—C3A_5—C2A_5	116 (3)
Cl14—Cu15—S1A_5	106.5 (9)	O1A_5—C3A_5—C2A_5	116 (2)
S1_5—Cu15—Cu3	50.4 (11)	C1_6—S1_6—Cu13	119.7 (6)
S1_4—Cu15—Cu3	103.52 (9)	C1_6—S1_6—Cu7	98.0 (5)
Cl14—Cu15—Cu3	129.66 (10)	Cu13—S1_6—Cu7	140.8 (3)
S1A_5—Cu15—Cu3	49.7 (13)	C1_6—S1_6—Cu8	109.6 (6)
S1_5—Cu15—Cu13	102.8 (10)	Cu13—S1_6—Cu8	94.8 (2)
S1_4—Cu15—Cu13	49.68 (10)	Cu7—S1_6—Cu8	80.91 (17)
Cl14—Cu15—Cu13	105.18 (10)	C1_6—S1_6—Cu14	104.4 (6)
S1A_5—Cu15—Cu13	104.0 (12)	Cu13—S1_6—Cu14	82.22 (17)
Cu3—Cu15—Cu13	122.34 (6)	Cu7—S1_6—Cu14	78.33 (16)
S1_5—Cu15—Cu12	102.6 (9)	Cu8—S1_6—Cu14	142.2 (3)
S1_4—Cu15—Cu12	48.12 (9)	C2_6—N1_6—H1A_6	109.5
Cl14—Cu15—Cu12	142.19 (10)	C2_6—N1_6—H1B_6	109.5
S1A_5—Cu15—Cu12	102.4 (11)	H1A_6—N1_6—H1B_6	109.5
Cu3—Cu15—Cu12	58.39 (5)	C2_6—N1_6—H1C_6	109.5
Cu13—Cu15—Cu12	90.58 (6)	H1A_6—N1_6—H1C_6	109.5
S1_5—Cu15—Cu14	47.1 (11)	H1B_6—N1_6—H1C_6	109.5
S1_4—Cu15—Cu14	103.29 (11)	C2_6—C1_6—S1_6	109.9 (11)
Cl14—Cu15—Cu14	100.56 (10)	C2_6—C1_6—H1D_6	109.7
S1A_5—Cu15—Cu14	48.2 (13)	S1_6—C1_6—H1D_6	109.7
Cu3—Cu15—Cu14	91.03 (6)	C2_6—C1_6—H1E_6	109.7
Cu13—Cu15—Cu14	59.11 (6)	S1_6—C1_6—H1E_6	109.7
Cu12—Cu15—Cu14	116.88 (6)	H1D_6—C1_6—H1E_6	108.2
Cl13—Cu16—Cl12	116.04 (13)	C1_6—C2_6—N1_6	105.8 (14)
Cl13—Cu16—Cl14	120.37 (12)	C1_6—C2_6—C3_6	109.7 (13)
Cl12—Cu16—Cl14	117.17 (13)	N1_6—C2_6—C3_6	103.0 (11)
Cl13—Cu16—Cl17	93.56 (10)	C1_6—C2_6—H2_6	112.6
Cl12—Cu16—Cl17	108.38 (10)	N1_6—C2_6—H2_6	112.6
Cl14—Cu16—Cl17	93.75 (10)	C3_6—C2_6—H2_6	112.6
Cl13—Cu16—Cu13	93.21 (9)	O1_6—C3_6—O2_6	128.3 (18)
Cl12—Cu16—Cu13	48.55 (7)	O1_6—C3_6—C2_6	116.3 (11)
Cl14—Cu16—Cu13	102.38 (8)	O2_6—C3_6—C2_6	115.4 (11)
Cl17—Cu16—Cu13	156.21 (9)	H4A_6—O4_6—H4B_6	105.8 (3)
Cu20—Cl17—Cu17	77.44 (9)	C1A_6—S1A_6—Cu13	75.8 (15)
Cu20—Cl17—Cu16	154.44 (13)	C1A_6—S1A_6—Cu7	137 (2)
Cu17—Cl17—Cu16	84.78 (10)	Cu13—S1A_6—Cu7	142.4 (15)
Cu18—Cl18—Cu17	82.21 (10)	C1A_6—S1A_6—Cu8	124 (2)
Cu19—Cl19—Cu17	81.85 (10)	Cu13—S1A_6—Cu8	95.0 (8)
Cu28—Cl20—Cu27	78.24 (9)	Cu7—S1A_6—Cu8	81.2 (6)
Cu21—Cl21—Cu18	67.43 (8)	C1A_6—S1A_6—Cu14	90.9 (17)
Cu28—Cl22—Cu21	88.70 (9)	Cu13—S1A_6—Cu14	82.8 (7)
Cu28—Cl22—Cu32^iv^	78.91 (9)	Cu7—S1A_6—Cu14	79.0 (6)
Cu21—Cl22—Cu32^iv^	146.01 (11)	Cu8—S1A_6—Cu14	143.6 (15)
Cu29—Cl23—Cu28	83.99 (10)	C2A_6—N1A_6—H1AA_6	109.5
Cu26—Cl24—Cu22	78.41 (9)	C2A_6—N1A_6—H1AB_6	109.5
Cu30—Cl25—Cu26	76.07 (9)	H1AA_6—N1A_6—H1AB_6	109.5
Cu30—Cl25—Cu26^v^	146.40 (12)	C2A_6—N1A_6—H1AC_6	109.5
Cu26—Cl25—Cu26^v^	76.32 (9)	H1AA_6—N1A_6—H1AC_6	109.5
Cu26—Cl27—Cu23	93.73 (11)	H1AB_6—N1A_6—H1AC_6	109.5
Cu32—Cl30—Cu25	99.77 (11)	C2A_6—C1A_6—S1A_6	106 (3)
Cu31—Cl31—Cu32	81.30 (10)	C2A_6—C1A_6—Cu13	154 (3)
Cu31—Cl31—Cu28^vi^	148.72 (13)	S1A_6—C1A_6—Cu13	58.4 (11)
Cu32—Cl31—Cu28^vi^	75.18 (9)	C2A_6—C1A_6—H1BA_6	110.5
Cu24—Cl32—Cu32	81.82 (10)	S1A_6—C1A_6—H1BA_6	110.5
Cl19—Cu17—Cl18	119.80 (12)	Cu13—C1A_6—H1BA_6	63.0
Cl19—Cu17—Cl17	118.19 (11)	C2A_6—C1A_6—H1BB_6	110.5
Cl18—Cu17—Cl17	116.48 (12)	S1A_6—C1A_6—H1BB_6	110.5
Cl19—Cu17—Cl14	102.26 (10)	Cu13—C1A_6—H1BB_6	95.5
Cl18—Cu17—Cl14	97.50 (10)	H1BA_6—C1A_6—H1BB_6	108.7
Cl17—Cu17—Cl14	93.63 (10)	N1A_6—C2A_6—C1A_6	101 (3)
Cl19—Cu17—Cu20	101.40 (9)	N1A_6—C2A_6—C3A_6	142 (4)
Cl18—Cu17—Cu20	94.54 (9)	C1A_6—C2A_6—C3A_6	104 (3)
Cl17—Cu17—Cu20	50.29 (7)	N1A_6—C2A_6—H2A_6	101.6
Cl14—Cu17—Cu20	143.34 (9)	C1A_6—C2A_6—H2A_6	101.6
Cl19—Cu17—Cu19	48.85 (7)	C3A_6—C2A_6—H2A_6	101.6
Cl18—Cu17—Cu19	103.89 (8)	O1A_6—C3A_6—O2A_6	129 (2)
Cl17—Cu17—Cu19	95.15 (8)	O1A_6—C3A_6—C2A_6	115.2 (14)
Cl14—Cu17—Cu19	150.15 (8)	O2A_6—C3A_6—C2A_6	115.3 (14)
Cu20—Cu17—Cu19	55.61 (4)	C1_7—S1_7—Cu30	107.0 (3)
Cl19—Cu17—Cu18	96.70 (8)	C1_7—S1_7—Cu29	119.8 (3)
Cl18—Cu17—Cu18	48.61 (7)	Cu30—S1_7—Cu29	89.79 (10)
Cl17—Cu17—Cu18	101.84 (8)	C1_7—S1_7—Cu22	101.1 (3)
Cl14—Cu17—Cu18	146.12 (8)	Cu30—S1_7—Cu22	73.59 (8)
Cu20—Cu17—Cu18	55.87 (4)	Cu29—S1_7—Cu22	138.90 (11)
Cu19—Cu17—Cu18	58.51 (4)	C1_7—S1_7—Cu21	108.8 (3)
S1_11—Cu18—S1_9	123.85 (11)	Cu30—S1_7—Cu21	136.47 (11)
S1_11—Cu18—Cl18	125.71 (12)	Cu29—S1_7—Cu21	93.35 (10)
S1_9—Cu18—Cl18	107.75 (11)	Cu22—S1_7—Cu21	75.88 (9)
S1_11—Cu18—Cl21	98.07 (9)	C2_7—N1_7—H1A_7	109.5
S1_9—Cu18—Cl21	93.08 (9)	C2_7—N1_7—H1B_7	109.5
Cl18—Cu18—Cl21	94.64 (10)	H1A_7—N1_7—H1B_7	109.5
S1_11—Cu18—Cu20	101.28 (8)	C2_7—N1_7—H1C_7	109.5
S1_9—Cu18—Cu20	50.72 (7)	H1A_7—N1_7—H1C_7	109.5
Cl18—Cu18—Cu20	98.45 (9)	H1B_7—N1_7—H1C_7	109.5
Cl21—Cu18—Cu20	143.74 (8)	C2_7—C1_7—S1_7	116.2 (7)
S1_11—Cu18—Cu21	97.71 (8)	C2_7—C1_7—H1AA_7	108.2
S1_9—Cu18—Cu21	49.99 (7)	S1_7—C1_7—H1AA_7	108.2
Cl18—Cu18—Cu21	130.42 (9)	C2_7—C1_7—H1AB_7	108.2
Cl21—Cu18—Cu21	52.36 (7)	S1_7—C1_7—H1AB_7	108.2
Cu20—Cu18—Cu21	94.60 (6)	H1AA_7—C1_7—H1AB_7	107.4
S1_11—Cu18—Cu19	50.05 (7)	C1_7—C2_7—N1_7	112.5 (7)
S1_9—Cu18—Cu19	103.05 (8)	C1_7—C2_7—C3_7	111.4 (9)
Cl18—Cu18—Cu19	106.13 (9)	N1_7—C2_7—C3_7	109.6 (7)
Cl21—Cu18—Cu19	148.03 (7)	C1_7—C2_7—H2_7	107.7
Cu20—Cu18—Cu19	57.48 (4)	N1_7—C2_7—H2_7	107.7
Cu21—Cu18—Cu19	121.01 (6)	C3_7—C2_7—H2_7	107.7
S1_11—Cu18—Cu17	101.91 (8)	O1_7—C3_7—O2_7	127.9 (9)
S1_9—Cu18—Cu17	100.34 (8)	O1_7—C3_7—C2_7	120.2 (9)
Cl18—Cu18—Cu17	49.18 (7)	O2_7—C3_7—C2_7	111.9 (8)
Cl21—Cu18—Cu17	143.70 (8)	C1_8—S1_8—Cu24	120.5 (4)
Cu20—Cu18—Cu17	59.92 (5)	C1_8—S1_8—Cu27	101.0 (4)
Cu21—Cu18—Cu17	150.31 (6)	Cu24—S1_8—Cu27	138.17 (14)
Cu19—Cu18—Cu17	60.32 (4)	C1_8—S1_8—Cu29	112.0 (4)
S1_12—Cu19—Cl19	121.19 (10)	Cu24—S1_8—Cu29	88.23 (11)
S1_12—Cu19—S1_11	125.74 (11)	Cu27—S1_8—Cu29	80.61 (10)
Cl19—Cu19—S1_11	110.75 (11)	C1_8—S1_8—Cu25	104.3 (4)
S1_12—Cu19—Cu20	52.02 (7)	Cu24—S1_8—Cu25	86.31 (10)
Cl19—Cu19—Cu20	106.47 (8)	Cu27—S1_8—Cu25	77.87 (9)
S1_11—Cu19—Cu20	100.58 (8)	Cu29—S1_8—Cu25	140.61 (13)
S1_12—Cu19—Cu23	96.96 (8)	C2_8—N1_8—H1A_8	109.5
Cl19—Cu19—Cu23	134.24 (9)	C2_8—N1_8—H1B_8	109.5
S1_11—Cu19—Cu23	50.44 (7)	H1A_8—N1_8—H1B_8	109.5
Cu20—Cu19—Cu23	117.31 (6)	C2_8—N1_8—H1C_8	109.5
S1_12—Cu19—Cu18	105.59 (8)	H1A_8—N1_8—H1C_8	109.5
Cl19—Cu19—Cu18	99.31 (8)	H1B_8—N1_8—H1C_8	109.5
S1_11—Cu19—Cu18	48.44 (7)	C2_8—C1_8—S1_8	113.3 (9)
Cu20—Cu19—Cu18	58.27 (4)	C2_8—C1_8—H1AA_8	108.9
Cu23—Cu19—Cu18	92.90 (5)	S1_8—C1_8—H1AA_8	108.9
S1_12—Cu19—Cu17	100.71 (8)	C2_8—C1_8—H1AB_8	108.9
Cl19—Cu19—Cu17	49.31 (7)	S1_8—C1_8—H1AB_8	108.9
S1_11—Cu19—Cu17	101.30 (8)	H1AA_8—C1_8—H1AB_8	107.7
Cu20—Cu19—Cu17	60.49 (5)	C1_8—C2_8—N1_8	110.4 (11)
Cu23—Cu19—Cu17	151.71 (6)	C1_8—C2_8—C3_8	111.0 (11)
Cu18—Cu19—Cu17	61.16 (4)	N1_8—C2_8—C3_8	111.9 (10)
S1_9—Cu20—S1_12	131.59 (10)	C1_8—C2_8—H2_8	107.8
S1_9—Cu20—Cl17	121.93 (10)	N1_8—C2_8—H2_8	107.8
S1_12—Cu20—Cl17	106.48 (9)	C3_8—C2_8—H2_8	107.8
S1_9—Cu20—Cu19	111.22 (8)	O1_8—C3_8—O2_8	121.3 (13)
S1_12—Cu20—Cu19	52.45 (8)	O1_8—C3_8—C2_8	121.7 (12)
Cl17—Cu20—Cu19	103.60 (8)	O2_8—C3_8—C2_8	116.9 (12)
S1_9—Cu20—Cu18	52.97 (8)	C1_9—S1_9—Cu20	116.2 (3)
S1_12—Cu20—Cu18	111.47 (9)	C1_9—S1_9—Cu27	110.3 (4)
Cl17—Cu20—Cu18	111.36 (9)	Cu20—S1_9—Cu27	90.28 (10)
Cu19—Cu20—Cu18	64.24 (5)	C1_9—S1_9—Cu18	107.3 (4)
S1_9—Cu20—Cu17	105.73 (9)	Cu20—S1_9—Cu18	76.31 (9)
S1_12—Cu20—Cu17	103.85 (9)	Cu27—S1_9—Cu18	142.24 (11)
Cl17—Cu20—Cu17	52.28 (8)	C1_9—S1_9—Cu21	103.4 (3)
Cu19—Cu20—Cu17	63.90 (5)	Cu20—S1_9—Cu21	138.21 (11)
Cu18—Cu20—Cu17	64.21 (5)	Cu27—S1_9—Cu21	87.75 (10)
S1_9—Cu20—Cu25	98.59 (8)	Cu18—S1_9—Cu21	80.08 (10)
S1_12—Cu20—Cu25	49.91 (8)	C2_9—N1_9—H1A_9	109.5
Cl17—Cu20—Cu25	122.20 (9)	C2_9—N1_9—H1B_9	109.5
Cu19—Cu20—Cu25	96.60 (5)	H1A_9—N1_9—H1B_9	109.5
Cu18—Cu20—Cu25	126.17 (5)	C2_9—N1_9—H1C_9	109.5
Cu17—Cu20—Cu25	153.01 (6)	H1A_9—N1_9—H1C_9	109.5
S1_7—Cu21—S1_9	125.47 (10)	H1B_9—N1_9—H1C_9	109.5
S1_7—Cu21—Cl22	103.97 (9)	C2_9—C1_9—S1_9	115.2 (6)
S1_9—Cu21—Cl22	112.63 (10)	C2_9—C1_9—H1D_9	108.5
S1_7—Cu21—Cl21	113.78 (10)	S1_9—C1_9—H1D_9	108.5
S1_9—Cu21—Cl21	99.74 (10)	C2_9—C1_9—H1DE_9	108.5
Cl22—Cu21—Cl21	98.17 (10)	S1_9—C1_9—H1DE_9	108.5
S1_7—Cu21—Cu22	51.90 (7)	H1D_9—C1_9—H1DE_9	107.5
S1_9—Cu21—Cu22	106.71 (8)	C1_9—C2_9—C3_9	110.2 (7)
Cl22—Cu21—Cu22	140.56 (8)	C1_9—C2_9—N1_9	113.5 (8)
Cl21—Cu21—Cu22	71.92 (7)	C3_9—C2_9—N1_9	108.8 (8)
S1_7—Cu21—Cu18	113.33 (8)	C1_9—C2_9—H2_9	108.1
S1_9—Cu21—Cu18	49.93 (8)	C3_9—C2_9—H2_9	108.1
Cl22—Cu21—Cu18	141.89 (8)	N1_9—C2_9—H2_9	108.1
Cl21—Cu21—Cu18	60.22 (7)	O1_9—C3_9—O2_9	124.9 (9)
Cu22—Cu21—Cu18	66.55 (4)	O1_9—C3_9—C2_9	116.5 (9)
S1_11—Cu22—S1_7	136.31 (10)	O2_9—C3_9—C2_9	118.3 (10)
S1_11—Cu22—Cl24	117.62 (11)	C1_10—S1_10—Cu31	102.0 (3)
S1_7—Cu22—Cl24	106.06 (10)	C1_10—S1_10—Cu30	119.2 (3)
S1_11—Cu22—Cu30	112.01 (8)	Cu31—S1_10—Cu30	138.57 (12)
S1_7—Cu22—Cu30	52.69 (7)	C1_10—S1_10—Cu24	110.4 (4)
Cl24—Cu22—Cu30	103.69 (8)	Cu31—S1_10—Cu24	79.16 (10)
S1_11—Cu22—Cu21	102.36 (8)	Cu30—S1_10—Cu24	89.92 (11)
S1_7—Cu22—Cu21	52.22 (7)	C1_10—S1_10—Cu23	107.6 (4)
Cl24—Cu22—Cu21	120.35 (8)	Cu31—S1_10—Cu23	79.26 (10)
Cu30—Cu22—Cu21	99.50 (6)	Cu30—S1_10—Cu23	84.02 (10)
S1_11—Cu22—Cu26	107.03 (8)	Cu24—S1_10—Cu23	139.33 (12)
S1_7—Cu22—Cu26	99.29 (7)	C2_10—N1_10—H1A_10	109.5
Cl24—Cu22—Cu26	50.68 (7)	C2_10—N1_10—H1B_10	109.5
Cu30—Cu22—Cu26	61.91 (4)	H1A_10—N1_10—H1B_10	109.5
Cu21—Cu22—Cu26	149.48 (6)	C2_10—N1_10—H1C_10	109.5
S1_11—Cu22—Cu23	50.00 (7)	H1A_10—N1_10—H1C_10	109.5
S1_7—Cu22—Cu23	110.93 (8)	H1B_10—N1_10—H1C_10	109.5
Cl24—Cu22—Cu23	114.70 (8)	C2_10—C1_10—S1_10	114.5 (6)
Cu30—Cu22—Cu23	64.71 (5)	C2_10—C1_10—H1D_10	108.6
Cu21—Cu22—Cu23	124.92 (5)	S1_10—C1_10—H1D_10	108.6
Cu26—Cu22—Cu23	71.40 (4)	C2_10—C1_10—H1DE_10	108.6
S1_11—Cu23—S1_10	130.74 (11)	S1_10—C1_10—H1DE_10	108.6
S1_11—Cu23—Cl27	107.60 (11)	H1D_10—C1_10—H1DE_10	107.6
S1_10—Cu23—Cl27	106.22 (11)	N1_10—C2_10—C3_10	106.6 (8)
S1_11—Cu23—Cl29	102.64 (11)	N1_10—C2_10—C1_10	112.5 (8)
S1_10—Cu23—Cl29	105.62 (11)	C3_10—C2_10—C1_10	109.3 (7)
Cl27—Cu23—Cl29	99.91 (11)	N1_10—C2_10—H2_10	109.5
S1_11—Cu23—Cu31	110.46 (8)	C3_10—C2_10—H2_10	109.5
S1_10—Cu23—Cu31	49.04 (7)	C1_10—C2_10—H2_10	109.5
Cl27—Cu23—Cu31	141.71 (9)	O1_10—C3_10—O2_10	125.8 (9)
Cl29—Cu23—Cu31	67.92 (9)	O1_10—C3_10—C2_10	120.8 (10)
S1_11—Cu23—Cu19	50.49 (7)	O2_10—C3_10—C2_10	113.4 (9)
S1_10—Cu23—Cu19	112.11 (8)	C1_11—S1_11—Cu22	114.0 (3)
Cl27—Cu23—Cu19	140.89 (10)	C1_11—S1_11—Cu18	113.2 (3)
Cl29—Cu23—Cu19	62.83 (8)	Cu22—S1_11—Cu18	90.15 (10)
Cu31—Cu23—Cu19	67.52 (5)	C1_11—S1_11—Cu23	105.6 (3)
S1_11—Cu23—Cu22	48.18 (7)	Cu22—S1_11—Cu23	81.82 (9)
S1_10—Cu23—Cu22	96.99 (8)	Cu18—S1_11—Cu23	140.22 (14)
Cl27—Cu23—Cu22	91.03 (8)	C1_11—S1_11—Cu19	107.0 (3)
Cl29—Cu23—Cu22	150.82 (10)	Cu22—S1_11—Cu19	138.06 (13)
Cu31—Cu23—Cu22	117.61 (6)	Cu18—S1_11—Cu19	81.51 (9)
Cu19—Cu23—Cu22	91.82 (5)	Cu23—S1_11—Cu19	79.07 (9)
S1_11—Cu23—Cu30	99.64 (8)	C2_11—N1_11—H1A_11	109.5
S1_10—Cu23—Cu30	46.90 (7)	C2_11—N1_11—H1B_11	109.5
Cl27—Cu23—Cu30	88.51 (8)	H1A_11—N1_11—H1B_11	109.5
Cl29—Cu23—Cu30	152.44 (11)	C2_11—N1_11—H1C_11	109.5
Cu31—Cu23—Cu30	89.21 (6)	H1A_11—N1_11—H1C_11	109.5
Cu19—Cu23—Cu30	123.60 (6)	H1B_11—N1_11—H1C_11	109.5
Cu22—Cu23—Cu30	53.64 (4)	C2_11—C1_11—S1_11	116.1 (7)
S1_8—Cu24—S1_10	128.15 (11)	C2_11—C1_11—H1AA_11	108.3
S1_8—Cu24—Cl32	122.91 (12)	S1_11—C1_11—H1AA_11	108.3
S1_10—Cu24—Cl32	108.09 (11)	C2_11—C1_11—H1AB_11	108.3
S1_8—Cu24—Cu31	104.05 (9)	S1_11—C1_11—H1AB_11	108.3
S1_10—Cu24—Cu31	50.24 (7)	H1AA_11—C1_11—H1AB_11	107.4
Cl32—Cu24—Cu31	104.50 (9)	C3_11—C2_11—N1_11	107.5 (8)
S1_8—Cu24—Cu32	107.70 (9)	C3_11—C2_11—C1_11	110.2 (10)
S1_10—Cu24—Cu32	97.64 (8)	N1_11—C2_11—C1_11	113.3 (8)
Cl32—Cu24—Cu32	49.49 (7)	C3_11—C2_11—H2_11	108.6
Cu31—Cu24—Cu32	62.58 (5)	N1_11—C2_11—H2_11	108.6
S1_8—Cu25—S1_12	130.93 (11)	C1_11—C2_11—H2_11	108.6
S1_8—Cu25—Cl30	103.93 (11)	O1_11—C3_11—O2_11	121.8 (12)
S1_12—Cu25—Cl30	108.56 (10)	O1_11—C3_11—C2_11	122.6 (10)
S1_8—Cu25—Cl28	105.84 (11)	O2_11—C3_11—C2_11	115.0 (11)
S1_12—Cu25—Cl28	105.10 (11)	C1_12—S1_12—Cu31	114.3 (3)
Cl30—Cu25—Cl28	97.69 (10)	C1_12—S1_12—Cu20	109.0 (3)
S1_8—Cu25—Cu27	50.11 (7)	Cu31—S1_12—Cu20	136.06 (11)
S1_12—Cu25—Cu27	109.11 (8)	C1_12—S1_12—Cu19	115.8 (4)
Cl30—Cu25—Cu27	142.27 (10)	Cu31—S1_12—Cu19	92.02 (11)
Cl28—Cu25—Cu27	70.49 (8)	Cu20—S1_12—Cu19	75.54 (9)
S1_8—Cu25—Cu31	98.45 (8)	C1_12—S1_12—Cu25	102.6 (4)
S1_12—Cu25—Cu31	47.78 (7)	Cu31—S1_12—Cu25	82.12 (10)
Cl30—Cu25—Cu31	88.80 (8)	Cu20—S1_12—Cu25	81.52 (10)
Cl28—Cu25—Cu31	152.41 (10)	Cu19—S1_12—Cu25	139.87 (11)
Cu27—Cu25—Cu31	118.57 (6)	C2_12—N1_12—H1A_12	109.5
S1_8—Cu25—Cu20	109.99 (8)	C2_12—N1_12—H1B_12	109.5
S1_12—Cu25—Cu20	48.57 (7)	H1A_12—N1_12—H1B_12	109.5
Cl30—Cu25—Cu20	146.00 (9)	C2_12—N1_12—H1C_12	109.5
Cl28—Cu25—Cu20	71.12 (9)	H1A_12—N1_12—H1C_12	109.5
Cu27—Cu25—Cu20	65.65 (5)	H1B_12—N1_12—H1C_12	109.5
Cu31—Cu25—Cu20	88.57 (5)	C2_12—C1_12—S1_12	114.9 (7)
Cl24—Cu26—Cl27	120.65 (11)	C2_12—C1_12—H1AA_12	108.5
Cl24—Cu26—Cl25	118.76 (10)	S1_12—C1_12—H1AA_12	108.5
Cl27—Cu26—Cl25	114.22 (11)	C2_12—C1_12—H1AB_12	108.5
Cl24—Cu26—Cl25^v^	96.60 (9)	S1_12—C1_12—H1AB_12	108.5
Cl27—Cu26—Cl25^v^	98.93 (10)	H1AA_12—C1_12—H1AB_12	107.5
Cl25—Cu26—Cl25^v^	99.93 (9)	N1_12—C2_12—C1_12	111.0 (8)
Cl24—Cu26—Cu30	98.60 (8)	N1_12—C2_12—C3_12	109.5 (8)
Cl27—Cu26—Cu30	94.15 (9)	C1_12—C2_12—C3_12	110.2 (9)
Cl25—Cu26—Cu30	51.27 (7)	N1_12—C2_12—H2_12	108.7
Cl25^v^—Cu26—Cu30	151.19 (8)	C1_12—C2_12—H2_12	108.7
Cl24—Cu26—Cu22	50.91 (6)	C3_12—C2_12—H2_12	108.7
Cl27—Cu26—Cu22	94.55 (8)	O1_12—C3_12—O2_12	126.1 (10)
Cl25—Cu26—Cu22	101.78 (7)	O1_12—C3_12—C2_12	118.2 (10)
Cl25^v^—Cu26—Cu22	146.94 (8)	O2_12—C3_12—C2_12	115.7 (9)
Cu30—Cu26—Cu22	55.85 (4)	H1A_13—O1_13—H1B_13	112 (5)
Cl24—Cu26—Cu26^v^	101.19 (7)		
			
Cu5—S1_1—C1_1—C2_1	173 (2)	Cu13—S1A_6—C1A_6—C2A_6	−157 (3)
Cu10—S1_1—C1_1—C2_1	89 (3)	Cu7—S1A_6—C1A_6—C2A_6	46 (4)
Cu8—S1_1—C1_1—C2_1	−10 (3)	Cu8—S1A_6—C1A_6—C2A_6	−70 (3)
Cu6—S1_1—C1_1—C2_1	−108 (3)	Cu14—S1A_6—C1A_6—C2A_6	121 (2)
S1_1—C1_1—C2_1—C3_1	166.7 (19)	Cu7—S1A_6—C1A_6—Cu13	−157 (2)
S1_1—C1_1—C2_1—N1_1	−74 (3)	Cu8—S1A_6—C1A_6—Cu13	86.5 (14)
C1_1—C2_1—C3_1—O1_1	140 (3)	Cu14—S1A_6—C1A_6—Cu13	−82.4 (8)
N1_1—C2_1—C3_1—O1_1	19 (3)	S1A_6—C1A_6—C2A_6—N1A_6	−81 (5)
C1_1—C2_1—C3_1—O2_1	−35 (3)	Cu13—C1A_6—C2A_6—N1A_6	−131 (6)
N1_1—C2_1—C3_1—O2_1	−156 (2)	S1A_6—C1A_6—C2A_6—C3A_6	128 (4)
Cu8—S1A_1—C1A_1—C2A_1	81 (6)	Cu13—C1A_6—C2A_6—C3A_6	79 (7)
Cu6—S1A_1—C1A_1—C2A_1	−24 (7)	N1A_6—C2A_6—C3A_6—O1A_6	77 (10)
Cu10—S1A_1—C1A_1—C2A_1	177 (5)	C1A_6—C2A_6—C3A_6—O1A_6	−154 (4)
Cu5—S1A_1—C1A_1—C2A_1	−103 (6)	N1A_6—C2A_6—C3A_6—O2A_6	−106 (10)
S1A_1—C1A_1—C2A_1—N1A_1	−78 (6)	C1A_6—C2A_6—C3A_6—O2A_6	23 (4)
S1A_1—C1A_1—C2A_1—C3A_1	147 (5)	Cu30—S1_7—C1_7—C2_7	110.9 (6)
C1A_1—C2A_1—C3A_1—O1A_1	107 (7)	Cu29—S1_7—C1_7—C2_7	11.1 (8)
N1A_1—C2A_1—C3A_1—O1A_1	−25 (7)	Cu22—S1_7—C1_7—C2_7	−173.0 (6)
C1A_1—C2A_1—C3A_1—O2A_1	−71 (8)	Cu21—S1_7—C1_7—C2_7	−94.3 (6)
N1A_1—C2A_1—C3A_1—O2A_1	157 (4)	S1_7—C1_7—C2_7—N1_7	−67.5 (9)
Cu5—S1_2—C1_2—C2_2	79.5 (10)	S1_7—C1_7—C2_7—C3_7	169.0 (7)
Cu2—S1_2—C1_2—C2_2	−24.2 (11)	C1_7—C2_7—C3_7—O1_7	129.1 (10)
Cu3—S1_2—C1_2—C2_2	−114.6 (9)	N1_7—C2_7—C3_7—O1_7	4.0 (14)
Cu12—S1_2—C1_2—C2_2	161.2 (9)	C1_7—C2_7—C3_7—O2_7	−50.7 (11)
S1_2—C1_2—C2_2—C3_2	163.3 (12)	N1_7—C2_7—C3_7—O2_7	−175.7 (9)
S1_2—C1_2—C2_2—N1_2	−73.6 (15)	Cu24—S1_8—C1_8—C2_8	−2.2 (11)
C1_2—C2_2—C3_2—O1_2	141 (2)	Cu27—S1_8—C1_8—C2_8	−176.5 (8)
N1_2—C2_2—C3_2—O1_2	14 (3)	Cu29—S1_8—C1_8—C2_8	99.3 (9)
C1_2—C2_2—C3_2—O2_2	−25 (3)	Cu25—S1_8—C1_8—C2_8	−96.3 (9)
N1_2—C2_2—C3_2—O2_2	−152 (2)	S1_8—C1_8—C2_8—N1_8	−71.9 (12)
Cu4—S1_3—C1_3—C2_3	−6.5 (11)	S1_8—C1_8—C2_8—C3_8	163.4 (9)
Cu7—S1_3—C1_3—C2_3	86.9 (9)	C1_8—C2_8—C3_8—O1_8	114.5 (17)
Cu2—S1_3—C1_3—C2_3	−88.6 (9)	N1_8—C2_8—C3_8—O1_8	−9 (2)
Cu6—S1_3—C1_3—C2_3	−174.8 (8)	C1_8—C2_8—C3_8—O2_8	−63.2 (18)
S1_3—C1_3—C2_3—N1_3	−64.4 (11)	N1_8—C2_8—C3_8—O2_8	173.0 (14)
S1_3—C1_3—C2_3—C3_3	174.3 (8)	Cu20—S1_9—C1_9—C2_9	−10.2 (10)
N1_3—C2_3—C3_3—O2_3	−3.6 (12)	Cu27—S1_9—C1_9—C2_9	90.7 (8)
C1_3—C2_3—C3_3—O2_3	119.2 (11)	Cu18—S1_9—C1_9—C2_9	−93.1 (8)
N1_3—C2_3—C3_3—O1_3	174.8 (8)	Cu21—S1_9—C1_9—C2_9	−176.7 (7)
C1_3—C2_3—C3_3—O1_3	−62.4 (12)	S1_9—C1_9—C2_9—C3_9	176.4 (8)
Cu15—S1_4—C1_4—C2_4	−3.7 (12)	S1_9—C1_9—C2_9—N1_9	−61.3 (10)
Cu10—S1_4—C1_4—C2_4	177.8 (9)	C1_9—C2_9—C3_9—O1_9	−57.1 (13)
Cu12—S1_4—C1_4—C2_4	92.3 (9)	N1_9—C2_9—C3_9—O1_9	177.9 (9)
Cu13—S1_4—C1_4—C2_4	−94.7 (10)	C1_9—C2_9—C3_9—O2_9	116.8 (11)
S1_4—C1_4—C2_4—C3_4	167.2 (10)	N1_9—C2_9—C3_9—O2_9	−8.2 (12)
S1_4—C1_4—C2_4—N1_4	−70.6 (12)	Cu31—S1_10—C1_10—C2_10	176.7 (7)
N1_4—C2_4—C3_4—O1_4	18.0 (18)	Cu30—S1_10—C1_10—C2_10	−8.0 (10)
C1_4—C2_4—C3_4—O1_4	141.5 (14)	Cu24—S1_10—C1_10—C2_10	93.9 (8)
N1_4—C2_4—C3_4—O2_4	−157.4 (12)	Cu23—S1_10—C1_10—C2_10	−100.9 (8)
C1_4—C2_4—C3_4—O2_4	−33.9 (18)	S1_10—C1_10—C2_10—N1_10	−61.7 (10)
Cu15—S1_5—C1_5—C2_5	−78 (3)	S1_10—C1_10—C2_10—C3_10	−179.8 (7)
Cu14—S1_5—C1_5—C2_5	21 (4)	N1_10—C2_10—C3_10—O1_10	−6.8 (12)
Cu3—S1_5—C1_5—C2_5	−164 (3)	C1_10—C2_10—C3_10—O1_10	115.0 (10)
Cu4—S1_5—C1_5—C2_5	114 (3)	N1_10—C2_10—C3_10—O2_10	175.3 (8)
S1_5—C1_5—C2_5—C3_5	161 (2)	C1_10—C2_10—C3_10—O2_10	−62.9 (12)
S1_5—C1_5—C2_5—N1_5	−73 (3)	Cu22—S1_11—C1_11—C2_11	73.4 (8)
C1_5—C2_5—C3_5—O1_5	−44 (3)	Cu18—S1_11—C1_11—C2_11	−27.8 (9)
N1_5—C2_5—C3_5—O1_5	−170 (2)	Cu23—S1_11—C1_11—C2_11	161.2 (7)
C1_5—C2_5—C3_5—O2_5	144 (3)	Cu19—S1_11—C1_11—C2_11	−115.7 (7)
N1_5—C2_5—C3_5—O2_5	18 (3)	S1_11—C1_11—C2_11—C3_11	162.7 (7)
S1A_5—C1A_5—C2A_5—N1A_5	−73 (3)	S1_11—C1_11—C2_11—N1_11	−76.8 (10)
S1A_5—C1A_5—C2A_5—C3A_5	166 (2)	N1_11—C2_11—C3_11—O1_11	−4.1 (18)
N1A_5—C2A_5—C3A_5—O2A_5	24 (4)	C1_11—C2_11—C3_11—O1_11	119.8 (14)
C1A_5—C2A_5—C3A_5—O2A_5	139 (3)	N1_11—C2_11—C3_11—O2_11	167.0 (12)
N1A_5—C2A_5—C3A_5—O1A_5	−158 (3)	C1_11—C2_11—C3_11—O2_11	−69.1 (14)
C1A_5—C2A_5—C3A_5—O1A_5	−43 (3)	Cu31—S1_12—C1_12—C2_12	83.5 (7)
Cu13—S1_6—C1_6—C2_6	−8.7 (14)	Cu20—S1_12—C1_12—C2_12	−104.3 (7)
Cu7—S1_6—C1_6—C2_6	−177.8 (11)	Cu19—S1_12—C1_12—C2_12	−21.6 (8)
Cu8—S1_6—C1_6—C2_6	99.0 (12)	Cu25—S1_12—C1_12—C2_12	170.4 (6)
Cu14—S1_6—C1_6—C2_6	−97.9 (12)	S1_12—C1_12—C2_12—N1_12	−69.9 (10)
S1_6—C1_6—C2_6—N1_6	−80.6 (15)	S1_12—C1_12—C2_12—C3_12	168.6 (7)
S1_6—C1_6—C2_6—C3_6	168.9 (11)	N1_12—C2_12—C3_12—O1_12	−3.2 (17)
C1_6—C2_6—C3_6—O1_6	129 (2)	C1_12—C2_12—C3_12—O1_12	119.2 (13)
N1_6—C2_6—C3_6—O1_6	17 (3)	N1_12—C2_12—C3_12—O2_12	175.4 (11)
C1_6—C2_6—C3_6—O2_6	−52 (3)	C1_12—C2_12—C3_12—O2_12	−62.3 (14)
N1_6—C2_6—C3_6—O2_6	−165 (2)		

Symmetry codes: (i) −*x*+1/2, *y*−1/2, −*z*+1; (ii) −*x*, *y*, −*z*+1; (iii) −*x*+1/2, *y*+1/2, −*z*+1; (iv) −*x*+1/2, *y*+1/2, −*z*; (v) −*x*+1, *y*, −*z*; (vi) −*x*+1/2, *y*−1/2, −*z*.

### Hydrogen-bond geometry (Å, º)

**Table:**

*D*—H···*A*	*D*—H	H···*A*	*D*···*A*	*D*—H···*A*
N1_1—H1*A*_1···Cl6	0.91	2.73	3.554 (19)	151
N1_1—H1*A*_1···Cl16	0.91	2.66	3.148 (16)	115
N1_1—H1*B*_1···Cl3^iii^	0.91	2.81	3.42 (2)	126
N1*A*_1—H1*AA*_1···Cl7	0.91	2.74	3.34 (4)	125
N1*A*_1—H1*AA*_1···Cl9	0.91	2.54	3.28 (3)	138
N1_2—H1*A*_2···Cl4	0.91	2.78	3.395 (15)	126
N1_2—H1*A*_2···O2*A*_5^iii^	0.91	2.48	3.04 (2)	120
N1_2—H1*C*_2···Cl5	0.91	2.19	3.101 (15)	175
N1_3—H1*A*_3···Cl10	0.91	2.69	3.592 (9)	171
N1_3—H1*B*_3···O2_3	0.91	2.13	2.605 (10)	112
N1_3—H1*C*_3···Cl2	0.91	2.53	3.306 (8)	143
N1_3—H1*C*_3···Cl11	0.91	2.68	3.228 (9)	120
N1_4—H1*B*_4···Cl15	0.91	2.44	3.199 (10)	141
N1_4—H1*B*_4···S1_4	0.91	2.82	3.300 (10)	114
N1_5—H1*B*_5···O2_12	0.91	2.36	3.14 (3)	144
N1_5—H1*C*_5···Cl11	0.91	2.76	3.46 (2)	134
N1_5—H1*C*_5···Cl13	0.91	2.49	3.24 (2)	139
N1*A*_5—H1*A*2_5···Cl2	0.91	2.74	3.46 (2)	137
N1*A*_5—H1*A*2_5···Cl11	0.91	2.42	2.99 (2)	121
N1*A*_5—H1*A*2_5···S1*A*_5	0.91	2.68	3.22 (4)	119
N1*A*_5—H1*A*3_5···Cl4^i^	0.91	2.82	3.49 (3)	132
N1_6—H1*B*_6···O3_6	0.91	1.92	2.70 (3)	142
N1_6—H1*C*_6···Cl16	0.91	2.53	3.311 (16)	145
N1_6—H1*C*_6···S1_6	0.91	2.89	3.330 (16)	111
N1*A*_6—H1*AC*_6···Cl11	0.91	2.74	3.60 (7)	158
N1_7—H1*B*_7···Cl23	0.91	2.77	3.435 (9)	131
N1_7—H1*B*_7···Cl26	0.91	2.58	3.315 (9)	138
N1_7—H1*C*_7···O1_13	0.91	1.99	2.803 (12)	148
N1_8—H1*C*_8···Cl26	0.91	2.51	3.370 (12)	158
N1_8—H1*C*_8···S1_8	0.91	2.83	3.286 (13)	112
N1_9—H1*A*_9···Cl20	0.91	2.85	3.585 (9)	139
N1_9—H1*A*_9···O1_10^iv^	0.91	2.12	2.796 (11)	130
N1_9—H1*B*_9···O4_6	0.91	2.01	2.86 (3)	154
N1_9—H1*C*_9···Cl17	0.91	2.79	3.348 (9)	121
N1_9—H1*C*_9···Cl28	0.91	2.45	3.211 (9)	142
N1_10—H1*B*_10···Cl25	0.91	2.77	3.239 (9)	113
N1_10—H1*B*_10···Cl26	0.91	2.57	3.363 (8)	146
N1_10—H1*C*_10···O2_9^vi^	0.91	2.15	2.856 (12)	134
N1_11—H1*A*_11···O1_13^v^	0.91	2.08	2.971 (13)	167
N1_11—H1*C*_11···Cl21	0.91	2.61	3.362 (10)	141
N1_11—H1*C*_11···Cl24	0.91	2.61	3.235 (9)	126
N1_12—H1*A*_12···Cl31	0.91	2.92	3.387 (9)	113
N1_12—H1*C*_12···Cl29	0.91	2.44	3.316 (9)	161
O4_6—H4*B*_6···Cl12	0.84 (1)	2.89 (10)	3.32 (3)	114 (8)
O4_6—H4*B*_6···O3_6	0.84 (1)	1.76 (7)	2.51 (4)	148 (10)
O1_13—H1*A*_13···Cl25	0.82 (3)	2.97 (3)	3.501 (9)	125 (2)

Symmetry codes: (i) −*x*+1/2, *y*−1/2, −*z*+1; (iii) −*x*+1/2, *y*+1/2, −*z*+1; (iv) −*x*+1/2, *y*+1/2, −*z*; (v) −*x*+1, *y*, −*z*; (vi) −*x*+1/2, *y*−1/2, −*z*.
